# Engineering Protein Nanoparticles Functionalized with an Immunodominant *Coxiella burnetii* Antigen to Generate a Q Fever Vaccine

**DOI:** 10.1021/acs.bioconjchem.3c00317

**Published:** 2023-09-08

**Authors:** Aaron Ramirez, Jiin Felgner, Aarti Jain, Sharon Jan, Tyler J. Albin, Alexander J. Badten, Anthony E. Gregory, Rie Nakajima, Algimantas Jasinskas, Philip L. Felgner, Amanda M. Burkhardt, D. Huw Davies, Szu-Wen Wang

**Affiliations:** ^†^Department of Chemical and Biomolecular Engineering, ^‡^Vaccine Research and Development Center, Department of Physiology and Biophysics, ^§^Department of Chemistry, ^∥^Department of Biomedical Engineering, ^⊥^Chao Family Comprehensive Cancer Center, and ^#^Institute for Immunology, University of California, Irvine, California 92697, United States; ¶ Department of Clinical Pharmacy, School of Pharmacy, University of Southern California, Los Angeles, California 90089, United States

## Abstract

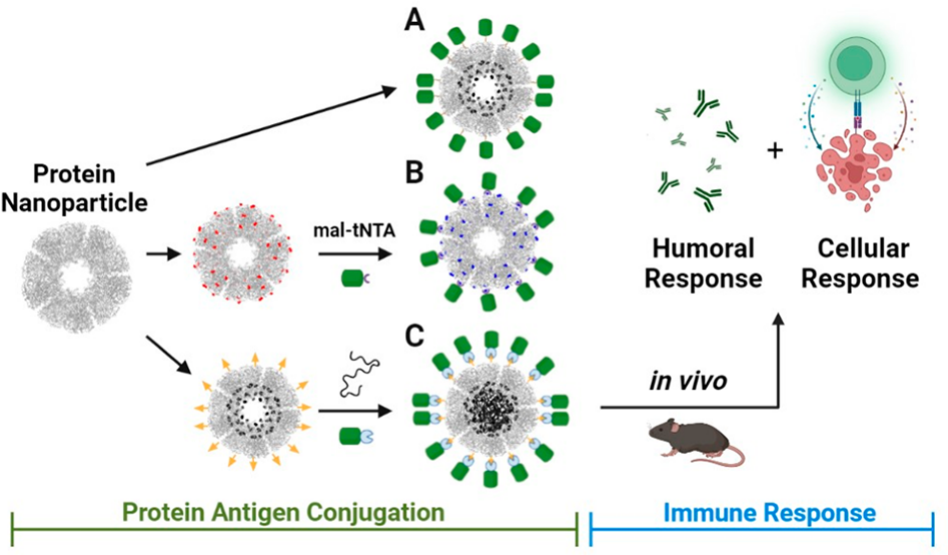

*Coxiella burnetii* is the causative agent of Q fever, for which there is yet to be an FDA-approved vaccine. This bacterial pathogen has both extra- and intracellular stages in its life cycle, and therefore both a cell-mediated (i.e., T lymphocyte) and humoral (i.e., antibody) immune response are necessary for effective eradication of this pathogen. However, most proposed vaccines elicit strong responses to only one mechanism of adaptive immunity, and some can either cause reactogenicity or lack sufficient immunogenicity. In this work, we aim to apply a nanoparticle-based platform toward producing both antibody and T cell immune responses against *C. burnetii*. We investigated three approaches for conjugation of the immunodominant outer membrane protein antigen (CBU1910) to the E2 nanoparticle to obtain a consistent antigen orientation: direct genetic fusion, high affinity tris-NTA-Ni conjugation to polyhistidine-tagged CBU1910, and the SpyTag/SpyCatcher (ST/SC) system. Overall, we found that the ST/SC approach yielded nanoparticles loaded with the highest number of antigens while maintaining stability, enabling formulations that could simultaneously co-deliver the protein antigen (CBU1910) and adjuvant (CpG1826) on one nanoparticle (CBU1910-CpG-E2). Using protein microarray analyses, we found that after immunization, antigen-bound nanoparticle formulations elicited significantly higher antigen-specific IgG responses than soluble CBU1910 alone and produced more balanced IgG1/IgG2c ratios. Although T cell recall assays from these protein antigen formulations did not show significant increases in antigen-specific IFN-γ production compared to soluble CBU1910 alone, nanoparticles conjugated with a CD4 peptide epitope from CBU1910 generated elevated T cell responses in mice to both the CBU1910 peptide epitope and whole CBU1910 protein. These investigations highlight the feasibility of conjugating antigens to nanoparticles for tuning and improving both humoral- and cell-mediated adaptive immunity against *C. burnetii*.

## Introduction

*Coxiella burnetii* is the Gram-negative intracellular bacterium that causes the life-threatening disease Q fever,^1−3^ and it has been classified by the US Center for Disease Control and Prevention as a potential bioterrorism agent due to its airborne transmission, highly infectious nature, and extreme resistance to environmental conditions.^1,3−5^ Q fever has an almost global distribution and can be found in a wide variety of animal reservoirs, with ruminants the most common.^6^ Human infections are often acquired from inhalation of contaminated aerosols resulting in an acute febrile illness, which can progress to pneumonia and hepatitis.^7^ In approximately 5% of cases, patients develop a potentially fatal chronic disease resulting in endocarditis, osteomyelitis, and chronic fatigue.^8^ In chronic forms of Q fever, that may arise weeks or years postinfection, long-term combination therapies are required to prevent death. Between 2007 and 2010, the largest known outbreak of Q fever occurred in The Netherlands resulting in >4000 cases.^9^ Of those identified as having chronic Q fever, mortality was 15.8%.^10^

Despite its pathogenic potential, an FDA-approved vaccine for this infectious agent is not yet available. A formalin-inactivated whole cell vaccine was previously licensed in Australia but was not approved in the US, and was discontinued due to the costs of production and required associated screening to prevent severe side effects in patients with previous exposure.^4,11,12^ Unlike typical bacterial pathogens, *C. burnetii* exhibits a tropism for professional immune system phagocytes (i.e., macrophages) and actively directs its own phagocytosis in order to reside within the terminal phagolysosomes of host cells in a favorable low pH environment, enabling its long-term survival and persistence.^1,13,14^ For this reason, a T lymphocyte response, in addition to an adequate antibody response, is considered necessary for eradication of the pathogen.^15−17^ In this investigation, we examine the ability to design and synthesize a *C. burnetii* vaccine using a protein nanoparticle (NP) platform to elicit both strong B and T cell responses. Although the advantages of NPs in vaccine development have been well-demonstrated,^18,19^ the design of antigen-conjugated nanoparticles for a Q fever vaccine has not yet been reported.

The protein NP utilized in this research is derived from the E2 subunit (E2) of the multienzyme complex, pyruvate dehydrogenase, sourced from *Geobacillus stearothermophilus*.^20,21^ E2 is a 60-subunit, self-assembling ∼25 nm dodecahedral scaffold with high stability that can be genetically engineered for precise chemical conjugation sites at the external surface and internal cavity.^20,22−26^ Our prior studies in developing cancer vaccines via a virus-mimetic strategy have demonstrated the utility of this scaffold for both adjuvant and antigen delivery.^27−31^ However, the application of this E2-based strategy for protection against bacterial pathogens has not yet been investigated. In this work, we utilize E2’s unique size, functional adaptability, and innate capability to elicit an antigen-specific immune response toward developing a prophylactic *C. burnetii* vaccine.

Proteomics and antigen-specific serological assays have identified the outer membrane protein CBU1910 as an immunodominant protein antigen of *C. burnetii*.^32−39^ For this reason, CBU1910 was chosen as the antigen for this prophylactic vaccine formulation. Unlike cancers, which can utilize peptide neoantigens in a vaccine to produce the desired anti-epitope T cell responses, infectious disease vaccines typically require the use of whole protein antigens to elicit both antibody and T cell responses.^40,41^ Protein antigens contain numerous immunogenic epitopes in native structural conformations, allowing for stronger antibody responses and broader adaptive immune responses.^40−42^ Although immunogenic peptide epitopes of *C. burnetii* have been identified and characterized for their potential use in vaccine development, application of these peptides in vaccines has not yet shown significant efficacy.^16,43−46^ More recently, vaccine formulations using *C. burnetii* protein antigens and triagonist adjuvants showed significant levels of protection for challenged animals, but to a lesser extent than the whole cell vaccine (which is not FDA approved).^47^ Thus, there is still a need for the development of a safer and efficacious prophylactic vaccine for *C. burnetii*.

**Figure 1:**
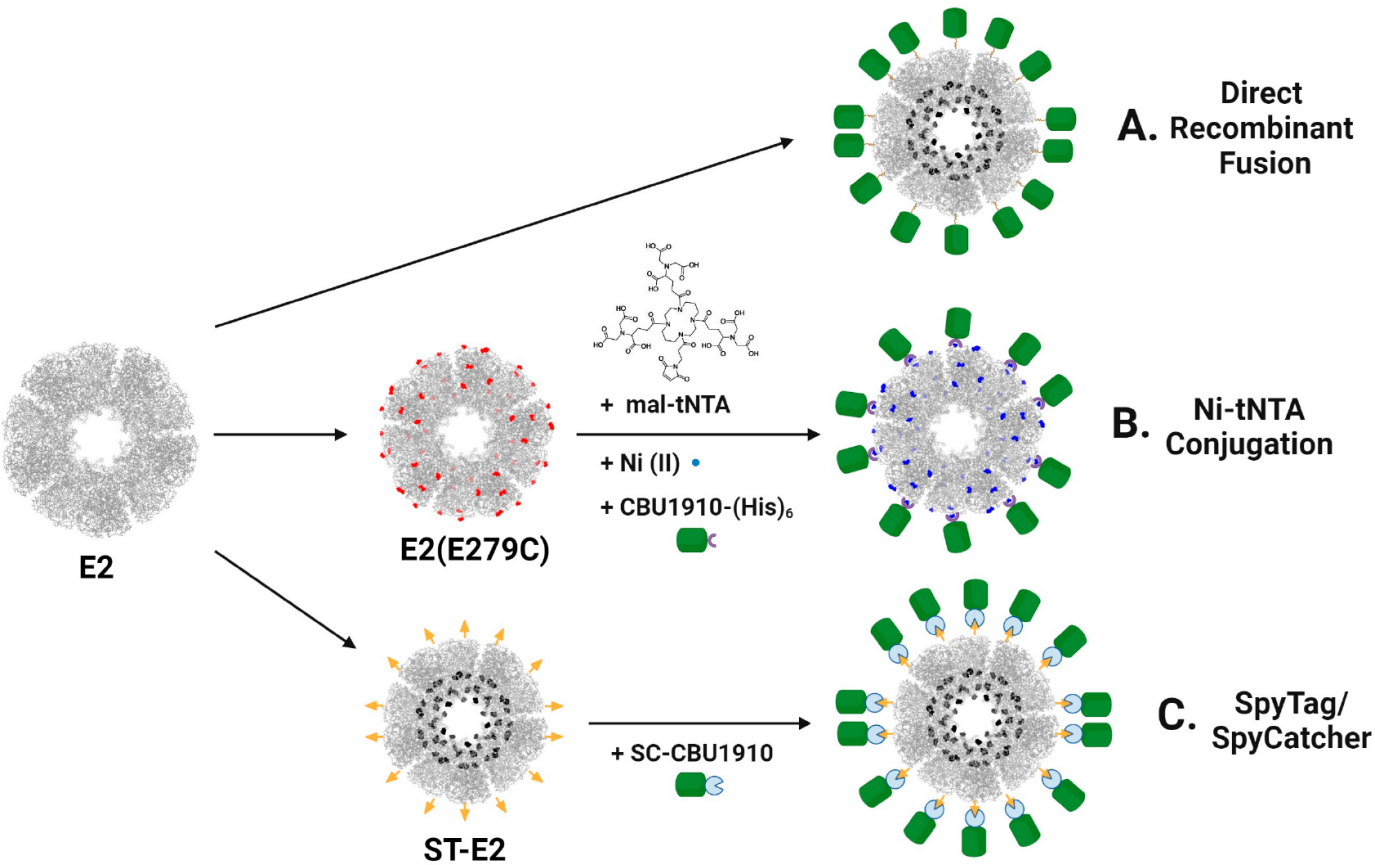
Overview of protein antigen–nanoparticle conjugation strategies. (A) Direct recombinant fusion of CBU1910 (green) onto E2 nanoparticles (gray). (B) Maleimide-tNTA-Ni linker chemistry on E2 nanoparticles (NPs) that contain surface cysteines (with the E279C mutation, red). His-tagged CBU1910 antigens are conjugated to the NP surface. (C) Assembly with a SpyTag/SpyCatcher system to conjugate ST-E2 and SC-CBU1910.

In this study, we investigated the integration of *C. burnetii* antigens onto the surface of the E2 NP. It is known that B cell activation and antibody responses are enhanced by a repetitive structural array on virus-like particles;^18,48,49^ however, there are currently limited options for conjugating protein antigens onto a NP surface while maintaining this consistent geometric orientation. Here, we examined three bioconjugation strategies that would enable a desired consistent antigen configuration: (1) direct recombinant fusion, (2) high affinity tris-NTA-Ni conjugation to polyhistidine-tagged (His-tag) antigen, and (3) the SpyTag(ST)/SpyCatcher(SC) system (Figure 1). Direct genetic fusion of protein antigens onto virus-like particles has shown some success with particular platforms and therefore was explored with the E2 protein nanoparticle; however, expression and correct folding into a soluble protein assembly needs to be empirically tested.^18,50−52^ The introduction of polyhistidine tags on recombinant proteins to bind to Ni-NTA-based matrices is a well-established protein purification methodology,^53−55^ and we previously applied this complexation-based approach in nanoparticle-mediated delivery of influenza hemagglutinin antigen;^56^ here, we used it as the basis for loading polyhistidine-tagged (His-tag) CBU1910 antigen onto E2 NPs. To attach protein antigens, covalently and modularly, onto the surface of E2 NPs, the versatile protein–protein conjugation method, SpyTag/SpyCatcher, was implemented.^57−62^ The adaptive immune response (i.e., antibody and T cell responses) to the most favorable NP construct was then examined to determine the prophylactic potential of the vaccine formulation.

## Results and Discussion

### Three Approaches Were Investigated for Loading *C. burnetii* Protein Antigen onto E2 Nanoparticles

We examined three strategies to attach the CBU1910 protein antigen to protein nanoparticles, as summarized in Figure 1 and described below. Table SI-1 lists the descriptions of each of the components and the corresponding abbreviations used in this work.

#### Direct Recombinant Fusion of CBU1910 onto E2 Nanoparticles

To investigate this loading strategy, we genetically fused CBU1910 to the N-terminus of a truncated E2 monomer. The wild-type form of the core E2 nanoparticle (dihydrolipoyl transacetylase) includes, on its N-terminus, a lipoyl domain and a peripheral subunit-binding domain, which enables association with the E1 and E3 proteins in the complex.^20,22,63^ In our studies, we distill this protein down to its structural dodecahedral core for application as a nanoparticle scaffold;^23−26,28^ however, based on the native structure, we hypothesized that other proteins with independent binding domains could be genetically fused to its N-terminus. We created two E2 mutants with different N-terminal linker lengths and 60 internal cavity cysteines [E2_152(D381C) and E2_158(D381C)]. Relative to the truncated E2(D381C) mutant used previously,^23,27,29−31^ the E2_152 and E2_158 mutants have an additional 20 and 14 amino acids of the wild-type protein sequence, respectively, added to their N-termini.^64,65^ Introduction of 60 internal cavity cysteines allows for adjuvant conjugation.^27,66^

The CBU1910 protein was recombinantly fused to E2 (Materials and Methods; Table SI-2), and the fusion proteins were expressed in *E. coli* (Figure SI-1). CBU1910 protein antigen fused to an E2 monomer was strongly expressed. However, these fusion proteins aggregated as inclusion bodies and were present only in the insoluble fraction, even under different expression conditions (e.g., lower temperatures, different induction conditions) (Figure SI-1). In contrast, the individual proteins (E2 monomers alone, CBU1910 alone) showed fractions which were soluble (Figure SI-1), with solubility linked to correct folding and nanoparticle assembly in prior studies.^23^ Under the conditions tested, the fused proteins (CBU1910-E2) could not be expressed as soluble proteins, suggesting misfolding and/or misassembly of the complex. For this reason, the two subsequent loading strategies focused on generating the two proteins separately, followed by conjugation together.

#### Using a tris-NTA-Ni Linker to Conjugate CBU1910 onto E2 Nanoparticles

**Figure 2:**
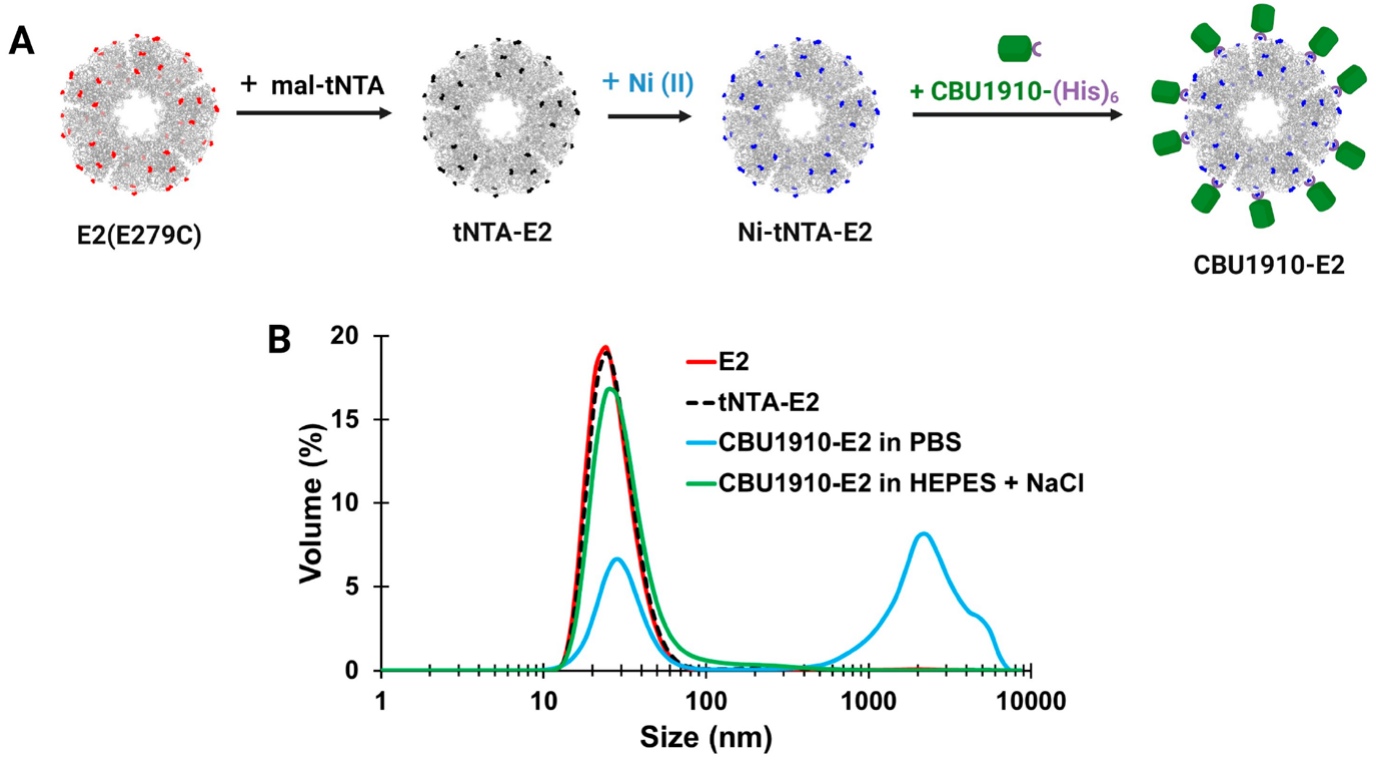
Conjugating CBU1910 onto E2 protein nanoparticles using a tris-NTA-Ni linker. (A) Schematic showing loading of CBU1910 of E2 nanoparticle via a tNTA-Ni linker. (B) Hydrodynamic diameters of E2 particles alone (E2), after linker conjugation (tNTA-E2), and CBU1910 loading on E2 (CBU1910-E2) in two different buffers. The NP component here is the E279C E2 mutant with cysteines displayed on the external surface.

To conjugate CBU1910 onto the surface of the E2 protein NP, we used an affinity strategy that we had developed for conjugating green fluorescent protein (GFP) and influenza hemagglutinin (HA).^56^ An E2 NP displaying 60 cysteines on its surface (E279C)^28^ allows for conjugation of a synthesized maleimide-tris-NTA (mal-tNTA) linker,^56^ which enables a His-tagged protein to couple to the NP. This protocol was performed for CBU1910 as shown in Figure 2A. Unlike GFP and HA conjugation, which remained soluble and physically stable over an extended period, conjugation of CBU1910-(His)_6_ to E2 yielded a mixture of single nanoparticles and aggregates of nanoparticles. Optimization of conjugation conditions (e.g., buffers, salts, surfactants) to yield non-aggregated nanoparticles was required, and these conditions are summarized in Figure SI-2. HEPES buffer at pH 7.3 with 360 mM NaCl was found to stabilize the nanoparticles and was used for subsequent purification and characterization steps. Free CBU1910-(His)_6_ was separated from E2-bound CBU1910 using size exclusion chromatography (SEC). Quantification of the number of E2-attached CBU1910 was determined to be 6 ± 3 per E2 nanoparticle.

Hydrodynamic diameters for E2, tNTA-E2, and CBU1910-E2 NPs were 27.3 ± 1.1 nm, 28.8 ± 2.2 nm, and 31.6 ± 4.0 nm, respectively, all of which fall in the size range shown to be beneficial for lymphatic system trafficking and antigen-presenting cell (i.e., dendritic cell and B cell) engagement (Figure 2B).^18,67^ The small increase in diameter for CBU1910-E2 was consistent with the relatively low number of CBU1910 on the surface of the E2 nanoparticle. To dose an adequate amount of antigen for an *in vivo* vaccine study, a 10-fold increase in the concentration was required; however, concentrating the formulation to this extent led to significant protein aggregation. Although conjugation of the CBU1910 antigen to the protein nanoparticle using a tNTA linker showed promise, this strategy showed limitations for this specific antigen that included low conjugation capacity and inconsistent physical stability. We note that this result is different than attachment of HA to E2, which had yielded reliable conjugation and stable, monodisperse particles, suggesting that these effects are highly antigen-dependent. Furthermore, the use of the maleimide-tNTA for surface conjugation via cysteine residues limits the use of cysteines internally for conjugation of immune-stimulating adjuvants.^27,29−31,66^

#### Using SpyTag(ST)/SpyCatcher(SC) to Conjugate CBU1910 onto E2 Nanoparticles

**Figure 3:**
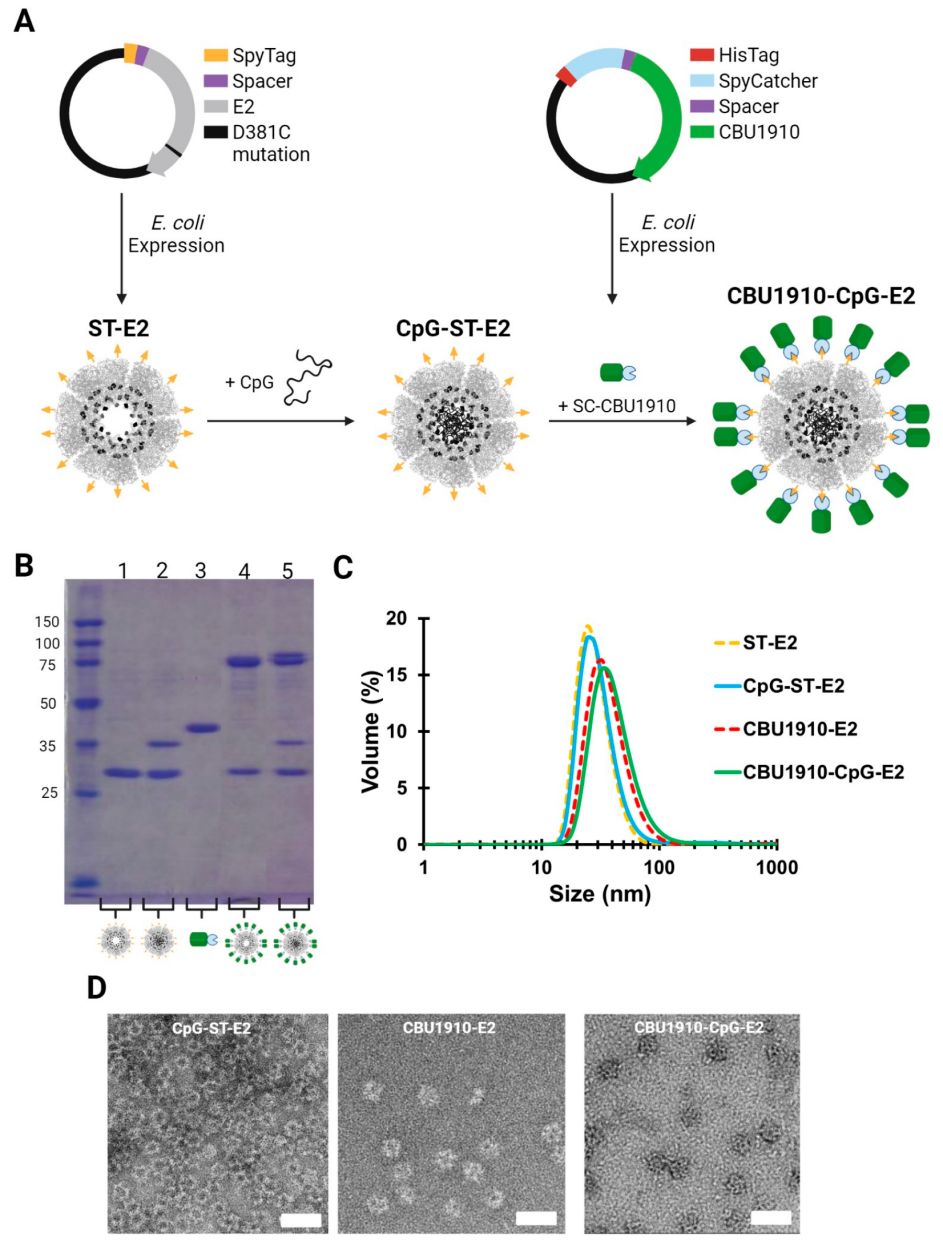
Conjugation of CBU1910 onto E2 nanoparticles using the SpyTag/SpyCatcher system. (A) Schematics of (top) plasmids for ST-E2 and SC-CBU1910 and (bottom) structure of expressed E2 with 60 SpyTags (yellow) on the surface and highlighted 60 cysteines (black) in the cavity and SpyCatcher-CBU1910 (green) fusion proteins. CpG1826 and SC-CBU1910 are conjugated onto ST-E2 to form CBU1910-CpG-E2. (B) SDS-PAGE of the nanoparticle components. Lanes: 1. ST-E2; 2. CpG-ST-E2; 3. SC-CBU1910; 4. CBU1910-E2; 5. CBU1910-CpG-E2. (C) Hydrodynamic diameters of E2 constructs after CpG and SC-CBU1910 conjugations. (D) Representative TEM images of the nanoparticles CpG-ST-E2, CBU1910-E2, and CBU1910-CpG-E2. Scale bar = 50 nm.

The SpyTag/SpyCatcher system^57^ was used to attach CBU1910 to the E2 nanoparticle using the strategy outlined in Figure 3A. The advantages of this approach include its stable covalent interaction and the ability to separately express both the antigen and the nanoparticle proteins prior to conjugation, which can circumvent protein expression challenges. SpyTag (ST) was genetically attached to the E2 nanoparticle, and SpyCatcher (SC) was genetically fused with the protein antigen CBU1910. We reasoned that coupling SpyCatcher to the antigen minimizes the amount of exposed SC after conjugation to the NP, which is likely favorable for reducing anti-SC immune responses.

The ST peptide was genetically fused to the N-terminus of E2 with a spacer sequence (Figure 3 and Figure SI-3). The E2 mutant D381C possessed 60 internal cavity cysteines, which would enable conjugation of adjuvant.^27,66^ Because a high-resolution protein structure of CBU1910 has not yet been determined, we used the protein folding prediction tool Alphafold2^68^ to predict the structure of CBU1910 (Figure SI-4). Based on this predicted structure of N-terminal truncated CBU1910 (to enable a soluble antigen),^47,69^ we decided to fuse SC to the N-terminus of CBU1910 (Figure 3; Figure SI-4). This ensured that when conjugated to the E2 nanoparticle, CBU1910 would be oriented in the same direction as when it is displayed on *C. burnetii*, exposing more relevant B cell epitopes.

The attachment of ST and SC to E2 and CBU1910, respectively, did not appear to decrease the expression levels or soluble protein amounts (Figure SI-3A). Therefore, we proceeded with purifying ST-E2(D381C) (henceforth referred to as ST-E2) and SC-CBU1910 for further characterization and studies. Both SDS-PAGE and mass spectrometry showed an expected molecular weight increase of ∼2.2 kDa (ST and spacer) for ST-E2 monomers (Figure SI-3B). The ST-E2 NP assembly yielded a hydrodynamic diameter of 29.2 ± 0.5 nm, which is slightly larger than the E2 diameter size of 27.8 ± 0.6 nm (Figure SI-3B), as expected. This is approximately 1 nm larger, which is consistent with previous literature estimates of ST on virus-like particles (VLPs).^58^ For SC-CBU1910, we achieved a >95% purity and the average molecular weight of ∼40.8 kDa, as determined by SDS-PAGE and mass spectrometry, which is consistent with SC fused to CBU1910 with a linker (Figure SI-3C).

The E2 protein NP platform, together with the ST/SC conjugation system, allows interior and exterior attachments designed for co-delivery of adjuvants and antigens, respectively. We conjugated the TLR-9 agonist, CpG1826, to the interior of the ST-E2 NP platform via an acid-labile BMPH linker (Figure 3A).^27^ Consistent with prior syntheses using E2, on SDS-PAGE the lower band on CpG-ST-E2 at ∼30 kDa shows the unconjugated ST-E2 monomer, and the band at ∼37 kDa supports the conjugation of one CpG molecule (∼7 kDa) to a ST-E2 monomer (Figure 3B). Quantification indicated 20.5 ± 1.5 CpG1826 molecules were encapsulated internally per 60-mer E2 NP, similar to previous E2 formulations.^27^ The average hydrodynamic diameter of the CpG-ST-E2 nanoparticles was 31.8 ± 1.4 nm (Figure 3C).

Although it is well-documented that the isopeptide bond formation between SpyTag and SpyCatcher is robust and reliable,^57,58,70−72^ conjugation of the SC-CBU1910 antigen onto the surface to ST-E2 required optimization to yield intact and monodisperse nanoparticles. As seen with other ST/SC VLP formulations, adjustments to reaction molar ratios, pH, ionic strength, and/or detergent concentrations were required to prevent precipitation/aggregation.^58,73−76^ A tabulated list of the optimization conditions and solubilizing additives (i.e., surfactants, buffers, and salts) can be found in Figure SI-5. From our investigation, we determined favorable reaction conditions to be a 1:0.5 molar ratio of ST-E2 (monomer):SC-CBU1910 at room temperature for 20 h with the addition of 0.08–0.0875% (w/v) SLS; this resulted in stable, monodisperse nanoparticles (Figure 3).

Size and antigen-to-nanoparticle ratios were then determined. When conjugated to SC-CBU1910, the ST-E2 monomer molecular weight increases by ∼41 to ∼71 kDa. As expected, when SC-CBU1910 is conjugated to CpG-ST-E2, two conjugate bands appear: one band is CBU1910-E2 monomers (∼71 kDa) and the other CBU1910-CpG-E2 monomers (∼78 kDa; both CBU1910 antigen and CpG conjugated onto E2) (Figure 3B). Quantification estimated that 29 ± 2 and 24 ± 2 SC-CBU1910 were conjugated to each ST-E2 and CpG-ST-E2 nanoparticle, respectively, out of a maximum possible number of 30 per nanoparticle (based on 1:0.5 molar ratio by monomer). CBU1910-E2 and CBU1910-CpG-E2 hydrodynamic diameters were 37.9 ± 1.9 nm and 43.6 ± 5.1 nm, respectively (Figure 3), with the size increase corresponding to the successful loading of antigens on the nanoparticles. Furthermore, TEM images confirmed intact monodisperse nanoparticles (Figure 3D). Because the ST/SC protein–protein conjugation system could be implemented to co-deliver protein antigen and adjuvant simultaneously, we used these stable nanoparticles (e.g., CBU-CpG-E2, CBU-E2) to evaluate their prophylactic vaccine potential.

### Attaching CBU1910 onto Nanoparticles Elicits Significantly Higher IgG Responses than Soluble CBU1910 Alone

**Figure 4:**
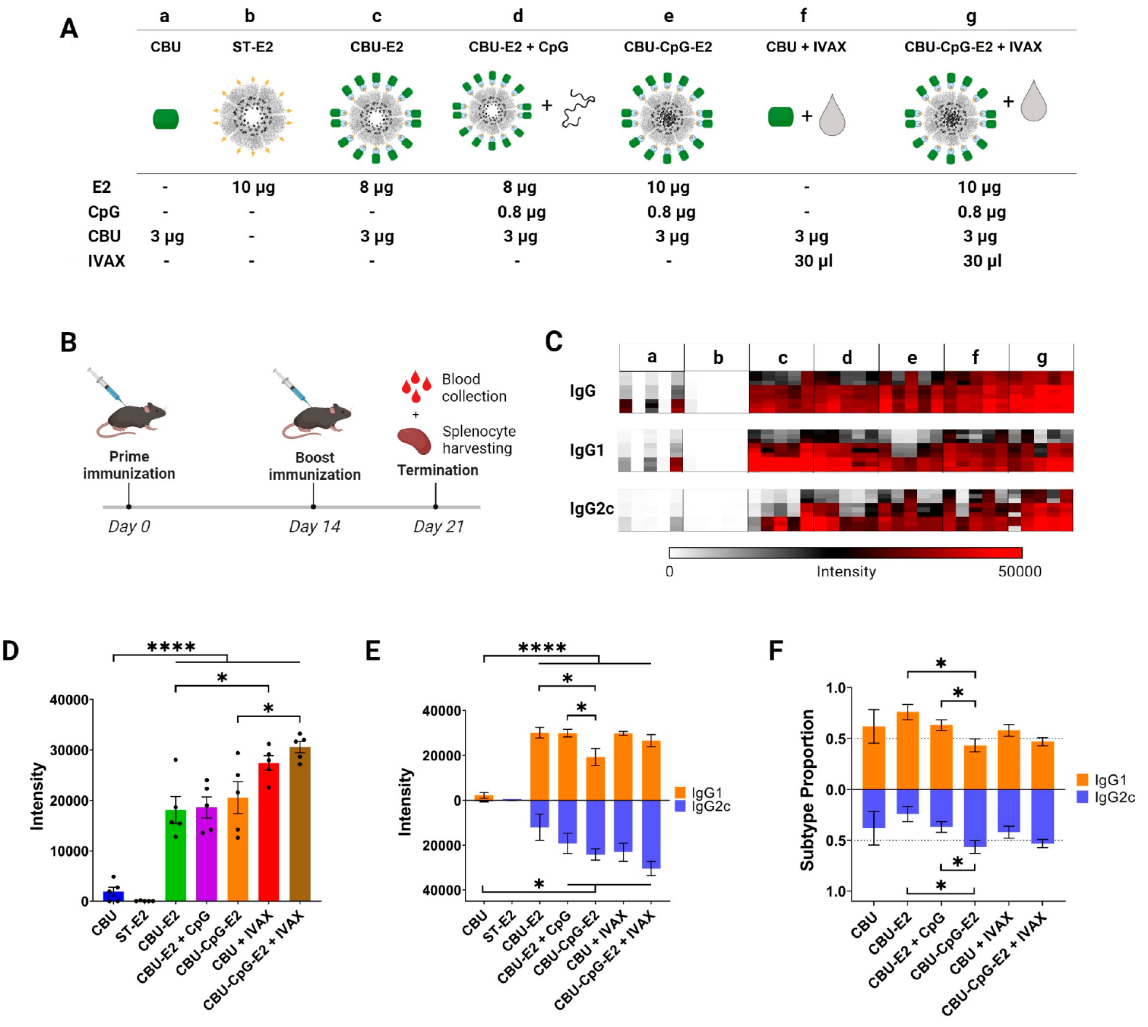
Antibody responses of protein antigen and nanoparticle formulations. (A) Table describing each formulation and its individual components. IVAX = 30 μL of Addavax emulsion + 1 nmol of CpG1018 + 3 nmol MPLA. (B) Schematic of prime/boost immunization schedule. (C) Heat map of antigen-specific antibody profiling using protein microarrays probed with plasma from day 21. CBU1910 was printed at three different concentrations of 0.1, 0.03, and 0.01 mg/mL (rows, bottom-to-top). Each column represents signal intensities of an individual mouse. (D) Total CBU1910-specific IgG in plasma on day 21. Quantification of data is shown in panel C (0.03 mg/mL array spots only). Each dot represents an individual mouse. (E) CBU1910-specific IgG1 and IgG2c in plasma on day 21. Quantification of data shown in panel C (0.03 mg/mL array spots only). (F) Calculated proportions of antibody subtypes in plasma on day 21 (intensities of IgG1 or IgG2c relative to [IgG1 + IgG2c]^47^). The subtype proportions of negative control ST-E2 was not applicable because IgG1 and IgG2 levels were at negligible background levels. Data in panels D, E, and F are presented as an average ± SEM of 5 mice per group (*n* = 5). Statistical significance was determined by one-way ANOVA followed by a Bonferroni multiple comparisons test. Two-tailed Student *t* tests were used in panel F. *p < 0.05, **p < 0.005, ****p < 0.0001. Abbreviations: CBU = CBU1910; CpG = CpG1826.

We investigated the antibody responses of different vaccine formulations containing the CBU1910 protein antigen, CpG1826, E2, and oil-in-water emulsion adjuvant, IVAX, after prime and boost immunization in mice (Figure 4A,B). The NPs generated using the ST/SC approach were selected for evaluation of immune responses due to their higher physical stability and protein antigen loading characteristics, as described above. Sera of each animal were evaluated using antigen microarrays to determine the antibody production elicited by each formulation (Figure 4C). The most striking result was seen when CBU1910 was displayed on E2 nanoparticles (CBU1910-E2), i.e., in the absence of adjuvants, which elevated the CBU1910-specific total IgG response significantly relative to soluble CBU1910 alone (Figure 4D).

It has been previously described that nanoparticle size and antigen display topography can play a crucial role in B cell engagement.^18,67^ Nanoparticles between ∼20–50 nm in diameter with antigen valences greater than ∼5 per nanoparticle and antigen spacing larger than ∼25 nm are reported to obtain effective B cell engagement.^18^ The CBU-E2 formulations used in this immunization study possess these characteristics and could be one explanation for obtaining >9 times increased IgG antibody response toward CBU by simply displaying the antigen on the nanoparticle platform (relative to unbound antigen). This also supports the premise that B cell activation can be augmented by the decoration of nanoparticles with repetitive epitopes; the repetitive geometry and spatial configuration of NP-attached antigens mimic natural pathogens such as viruses, which can yield strong innate adjuvating outcomes by improving uptake by antigen-presenting cells and enabling binding and simultaneous activation of multiple B cell receptors.^18,77^

### Adjuvant, Either Co-administered in Solution or Encapsulated within the Nanoparticle, Increases Anti-CBU1910 Titers

The IgG responses obtained with CpG-E2-based formulations were comparable to the positive control oil-in-water emulsion adjuvant, IVAX, a combination adjuvant consisting of AddaVax (a squalene-based adjuvant), monophosphoryl-lipid A (MPLA), and CpG1018, that has been shown to induce broadly reactive responses to influenza HA proteins^78^ (Group f, Figure 4D). Our previous *in vitro* studies using CpG-E2 nanoparticles demonstrated that once taken up by a cell, encapsulated CpG can be released from the nanoparticle in an acidic environment and activate mouse bone marrow-derived dendritic cells.^27^ Furthermore, encapsulated CpG was shown to activate these cells at significantly lower concentrations than unbound CpG, indicating the need for bioconjugation of CpG to the nanoparticle.^27^ Other studies have also shown that alternative types of nanoparticles which simultaneously deliver both conjugated CpG and antigen can increase the immunogenicity and immune response mounted against the target antigen.^29,30,79−81^ Thus, to deliver adjuvants more precisely to immune cells involved in adaptive immunity, such as dendritic cells, CpG was encapsulated within the nanoparticle in an approach that increases uptake efficiency of CpG and the dose of CpG that an individual cell receives upon endocytosing a nanoparticle versus free unbound CpG. The effects of CpG adjuvant and its delivery covalently encapsulated within the E2 NP (CBU-CpG-E2) or co-administered with the E2 NP by mixing only (CBU-E2 + CpG) showed comparable results on total IgG responses (Groups c–e, Figure 4D). Addition of IVAX to the CBU1910-CpG-E2 formulation further increased the overall anti-CBU1910 IgG response (Figure 4D, Group g).

### E2 Formulations That Contained an Adjuvant Elicited More Balanced IgG1/IgG2c Antibody Responses

Antibody class switching to IgG1 and IgG2c is associated with the cytokine profiles released from Th2 and Th1 lymphocytes, respectively. Th2 responses are described by B cell proliferation, antibody production, and induction of IgG1 antibodies.^82,83^ Th1 responses are characterized by the activation of antigen-presenting cells, stimulation of T cells, and induction of IgG2c antibodies.^82−84^ Thus, IgG1 and IgG2c production can be used as indicators of Th2 and Th1 responses, respectively. Profiles of the IgG1 and IgG2c antibody responses suggest modulation capabilities that depend on the adjuvant used and whether it was loaded in the E2 nanoparticle (Figure 4E,F). Soluble CBU1910 (Group a) elicited a very weak total IgG response that was slightly skewed toward IgG1 (Th2). Loading CBU1910 onto nanoparticles (CBU1910-E2, Group c) significantly increased total IgG (predominantly IgG1 (Th2)), with some measurable IgG2c (Th1) isotype switched antibodies, suggesting that the E2 NP may have some inherent Th1 skewing properties in the absence of any TLR agonists. The addition of soluble CpG1826 to CBU1910-E2 particles (CBU1910-E2 + CpG1826; Group d) further increased the shift toward a Th1 response as expected, although few of these shifts were significantly different from CBU1910-E2 alone and did not significantly alter IgG1/IgG2c ratios. Uniquely, when CpG1826 was internally conjugated to the E2 vaccine particle (CBU1910-CpG1826-E2; Group e), the total IgG amount did not significantly change, but the nature of the response shifted toward a Th1 response, as indicated by a more balanced IgG1/IgG2c ratio, compared to soluble or no CpG adjuvant addition (Figure 4E,F). Our data show that nanoparticles loaded with CpG elicited a more balanced IgG1/IgG2c response. Addition of IVAX to the CBU1910-CpG-E2 nanoparticle slightly increased IgG1 and IgG2c responses without greatly affecting the subtype balance conferred by the E2 formulation itself.

### Immunization with *C. burnetii* Antigen Loaded onto E2 Nanoparticles Increased Antigen-Specific IFN-γ Secretion

**Figure 5:**
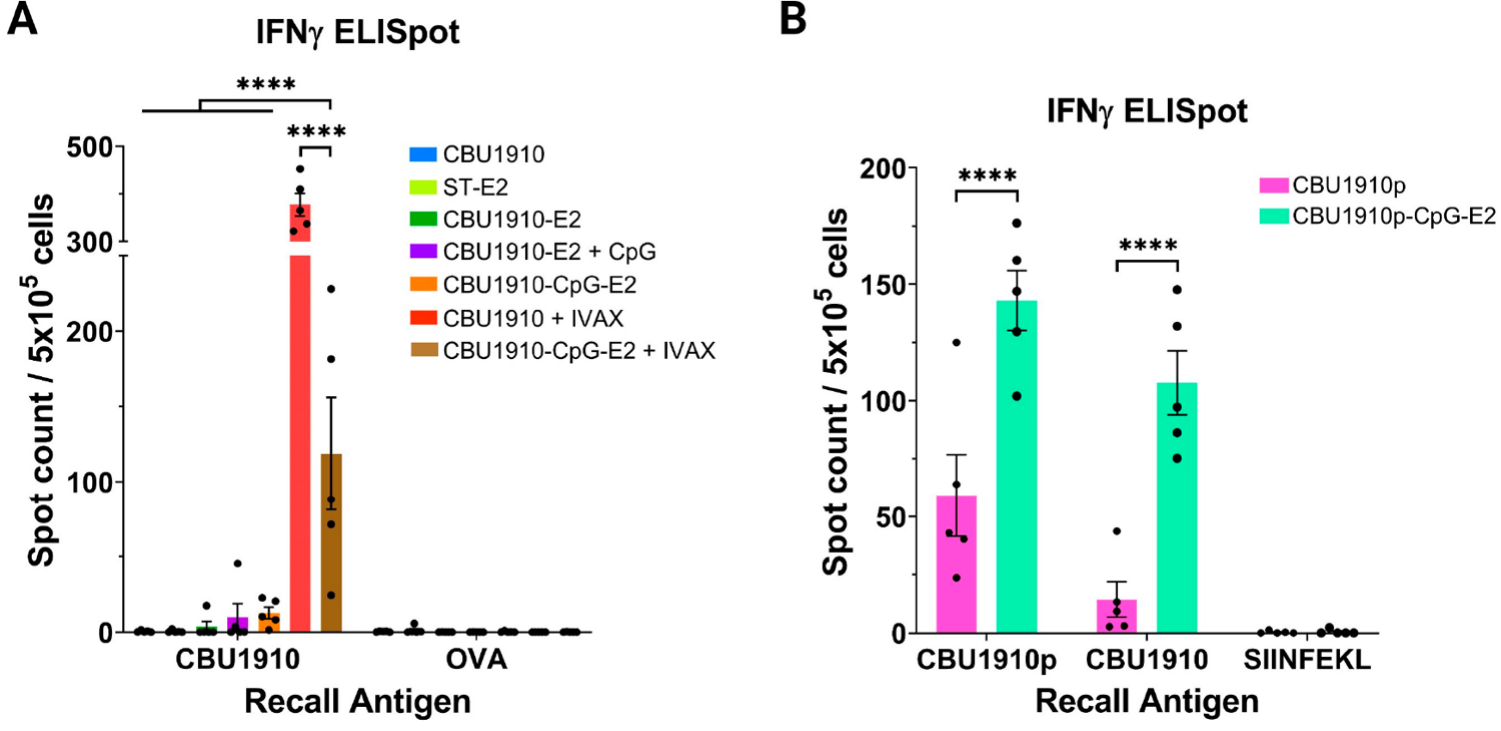
ELISpot analysis of splenocytes after immunizations with CBU1910 (protein) and CBU1910p (peptide epitope) formulations. (A) Summary of the average ELISpot data from mice immunized with different CBU1910 and E2 formulations. Splenocytes of immunized groups were pulsed *ex vivo* with relevant protein (CBU1910) or irrelevant protein (OVA) and analyzed for antigen-specific IFN-γ secretion. (**B**) Summary of the averaged ELISpot data from mice immunized with CBU1910p-CpG-E2 and CBU1910p alone. Splenocytes of immunized groups were pulsed *ex vivo* with a relevant peptide or protein (CBU1910p or CBU1910) or irrelevant peptide (SIINFEKL) and analyzed for antigen-specific IFN-γ secretion. Data is presented as an average ± SEM of 5 biological replicates. Statistical significance was determined by two-way ANOVA followed by a Bonferroni multiple comparisons test. ****p < 0.0001.

The more balanced IgG1/IgG2c antibody ratio elicited by CBU1910-CpG-E2 suggests that T cell responses toward the CBU1910 antigen are likely to be produced. However, when we examined the effector T cell response, we found that antigen-specific IFN-γ responses (which corresponds to an IgG2c/Th1 response) were low in mice that were administered CBU1910-CpG-E2 alone (Figure 5A and Figure SI-6). The addition of IVAX to this formulation significantly increased the level of IFN-γ secretion. This observation was not expected, given the similar levels of antigen-specific IgG2c production after immunization with the CBU1910-CpG-E2 nanoparticle, both with and without IVAX. The unexpectedly low IFN-γ response from formulations that induced IgG2c/Th1 responses compared to IVAX, such as CBU1910-CpG-E2, may have resulted from cytokines other than IFN-γ (including IL-2, IL-10, or TNF-α) driving the IgG2c response, or a different temporal release of IFN-γ (assayed after 18 h of restimulation with antigen, only). Further experiments would be necessary to test these hypotheses. Another reason for this discrepancy could stem from the TLR-4 agonist (MPLA) and TLR-9 agonist (CpG1018) present in IVAX. It has been shown that simultaneous stimulation of cell-surface and endosomal TLR receptors can cause synergistic increases in the activating/inflammatory immune response, and the soluble delivery of the adjuvants may cause a more systemic initial innate immune response.^85,86^ This differs from the delivery of CpG via a nanoparticle, which avoids non-specific systemic immune system stimulation due to encapsulation and increases uptake by antigen presenting cells (i.e., dendritic cells).^27^ Furthermore, previous studies that co-delivered peptide antigens and adjuvant for cancer vaccines resulted in dosages of ∼2–5 μg of CpG and ∼2–5 μg of T cell immunogenic peptide epitopes;^29−31^ however, for this protein antigen vaccine, both the CpG and immunodominant T cell epitope dosage were nearly an order of magnitude less than typically dosed for the peptide formulations of the cancer studies.

To determine if an E2 formulation was capable of inducing a strong antigen-specific T cell response toward *C. burnetii*, we examined if a peptide antigen (rather than protein antigen) coupled to the E2 NP could increase the cell-mediated response. Peptide antigens were conjugated to the NP using a linker and characterized, as described in the Supporting Information. Interestingly, we observed that immunizing with the CBU1910p-CpG-E2 formulation, an E2 nanoparticle loaded with the immunodominant CD4 T cell epitope peptide of CBU1910, HYLVNHPEVLVEASQ (CBU1910p), not only generated a higher frequency of CBU1910p-specific T cells, compared to soluble CBU1910p immunization, but also induced strong CBU1910-specific IFN-γ secretion (Figure 5B and Figure SI-6). This confirms that this peptide is a bona fide epitope generated by the natural processing of the whole CBU1910 antigen. This CBU1910p formulation allows for the delivery of ∼50-fold more of the immunodominant T cell epitope and ∼6-fold more of CpG than the CBU1910-bound NP formulation (Figure SI-7). These data suggest that using this epitope may be sufficient to elicit a strong cellular immune response toward the immunodominant protein antigen of *C. burnetii*, CBU1910. Given the strong antibody and T cell responses observed from the CBU1910-based and CBU1910p E2 NP formulations, respectively, a potential co-administration of the two warrants further investigations.

## Conclusions

We investigated three methods of conjugating the *C. burnetii* immunodominant protein antigen, CBU1910, to a protein nanoparticle: genetic fusion, tris-NTA-Ni linker, and ST/SC. We determined that ST/SC yielded vaccine nanoparticles with the highest antigen loading, had the capacity to internally encapsulate adjuvant, and could be formulated to yield stable, monodisperse particles. We engineered this platform, ST-E2(D381C), to allow for the simultaneous packing of Th1-skewing adjuvant, CpG, within the interior of the nanoparticle and displaying of protein antigen, CBU1910, on its surface. By displaying the antigen on the nanoparticle only, i.e., in the absence of CpG adjuvant, significant increases in antigen-specific IgG antibodies were observed in immunized mice compared to soluble antigen alone. The addition of an encapsulated or co-administered adjuvant balanced the IgG1/IgG2c antibody profile, which suggests induction of both Th1 (associated with cellular immunity) and Th2 (associated with humoral immunity) responses.

Both antibodies and T cells have been shown to contribute toward host-mediated protection against *C. burnetii* infection. While antibodies play an important role during the early stages of extracellular infection, T cell activation is vital for clearance of intracellular bacteria.^87^ Of particular significance are Th1 cells, identified by class switched B cells to produce IgG2c and IFN-γ positive CD4 T cells. Th1 cells contribute toward immunity by polarizing macrophages toward an M1 phenotype that is less permissive for intracellular *C. burnetii*.^88^ Th1 cells also support the activation of cytotoxic T cells, which target infected cells and aid in the formation of protective granulomas that surround parasitized cells. Moreover, Th1 cells contribute to long-term immunity through the generation of memory responses. A balance between antibody mediated and Th1 mediated immunity is essential for effective protection against *C. burnetii*. To confirm the presence of cell-mediated immunity more directly, we show that vaccination with a CBU1910 CD4 epitope peptide conjugated to E2 elicits robust Th1 responses, as measured via the T cell recall assay with IFN-γ ELISpot. This peptide-conjugation strategy has clear potential to confer protection against a pathogen with both extra- and intracellular stages to its life cycle, such as *C. burnetii*, which requires activation of both humoral and cellular arms of the immune system to grant protection.

## Materials and Methods

### Materials

All buffer and cloning reagents were purchased from Fisher Scientific unless otherwise noted. All cloning enzymes were purchased from New England Biolabs (NEB), unless otherwise noted. DH5α and BL21(DE3) *E. coli* were used for general cloning and expression studies, respectively. DNA minipreps and gel extractions were performed with the QIAprep Spin Miniprep Kit (Qiagen) and GeneJET Gel Extraction Kit (Thermo Fisher Scientific), respectively. DNA primers were synthesized and ordered from Integrated DNA Technologies (IDT). CloneJET PCR cloning kit (Thermo Fisher Scientific) was used for all polymerase chain reactions (PCRs). Plasmid pET11a was used as the expression vector for all protein constructs.

### Construction of CBU1910-E2 Fusion Protein Mutants

Previously established E2 mutants E2_152 and E2_158 were used to engineer CBU1910-E2 fusion constructs.^64,65^ D381C is an E2 mutation that introduces 60 cysteines to the internal cavity of the nanoparticle, allowing for internal conjugation.^23,27^ To introduce the D381C mutation to E2_158 and E2_152 via site directed mutagenesis (SDM)^89,90^ the forward primer: 5′-/5Phos/GCCGATCGTTCGTTGCGGTGAAATCGTTGC-3′ and reverse primer: 5′-/5Phos/TTTTCGGCTATACGACCAATACCCAG-3′ were used. To introduce the DNA cut sites required for ligation to the N-terminus of E2 mutants, Nde1 and Nhe1 cut sites were introduced to the N-terminus DNA coding region and C-terminus DNA coding region, respectively, of CBU1910 using the forward primer: 5′-CATATGCACCATCACCATCACCATCCGCAGCAAGTCAAAGACATTCAG-3′ and reverse primer: 5′-GCTAGCTTAGCCGCCGGTTTCCGG-3′. The plasmid encoding the CBU1910 protein (with its signal peptide deleted and portion of N-terminus truncated) was previously synthesized by GenScript Biotech^47,69^ and was used as the DNA template for all genetic engineering of the protein antigen.

A standard Phusion High-Fidelity DNA polymerase protocol was used for PCRs. These reactions were performed in a thermal cycler using a 30 s denaturation step at 98 °C, followed by 30 cycles of 15 s at 98 °C, 15 s at 58 °C (E2 D381C mutation) or 53 °C (CBU1910), and 7 min (E2 D381C mutation) or 45 s (CBU1910) at 72 °C, with a final step of 10 min at 72 °C. The CBU1910 gene was then ligated via the Nde1/Nhe1 sites of a pET11a vector that contained the E2 gene between Nhe1/BamH1. Sequencing was performed by GeneWiz/Azenta, and DNA and protein sequences are given in the Supporting Information.

### Expression of CBU1910-E2 Fusion Protein Mutants

The CBU1910-E2 fusion protein was expressed in a similar fashion to previous mutants described.^27,29,30^ Expression studies were performed for each mutant and controls. Proteins were expressed in BL21(DE3) *E. coli* via 1 mM IPTG induction. After induction for 3 h at 37 °C, cells were pelleted and stored at −80 °C. Cells were thawed and lysed by vortexing with glass beads. Soluble and insoluble lysates were centrifuged at 18000 × *g* for 15 min and analyzed using SDS-PAGE for molecular weight and soluble:insoluble ratios.

### Conjugation of mal-tNTA to E2 (E279C)

E2 (E297C) is an E2 mutant that displays 60 cysteines on its surface that can be used for thiol-based functionalization.^28^ We have reported the generation of tNTA-E2 nanoparticles previously.^56^ Purified E2 (E279C) in 20 mM HEPES and 100 mM NaCl (pH 7.3) was incubated with an 8.5× molar excess of TCEP (Thermo Fisher Scientific; dissolved in Milli-Q water). A 10× molar excess of maleimido cyclic tris-NTA (mal-tNTA) (diluted to 4 mg/mL in DMF) was added to the E2 and incubated at room temperature for 2 h and then at 4 °C overnight. Unreacted mal-tNTA, DMF, and TCEP were removed using Zeba spin desalting columns in 20 mM HEPES and 100 mM NaCl. Conjugation efficiency and characterization were determined using SDS-PAGE and mass spectrometry (Xevo G2-XS QTof) (Figure SI-8). The hydrodynamic diameter of the purified constructs was analyzed by dynamic light scattering (DLS) (Malvern Zetasizer Nano ZS).

### Attachment of His_6_-Tagged CBU1910 to tNTA-E2

Attachment of CBU1910-(His)_6_ to tNTA-E2 nanoparticles followed similar procedures established using other protein-(His)_n_ antigens.^56^ Briefly, a 10× molar excess of NiCl_2_ was incubated with tNTA-E2 for 2 h at room temperature and subsequently purified from unchelated Ni using Zeba spin desalting columns into 20 mM HEPES + 360 mM NaCl buffer pH 7.3. To test optimal conjugation, varying molar ratios of (His)_6_-tagged CBU1910 (previously synthesized^47^) were added to the Ni-tNTA-E2 and incubated at room temperature for 2 h. The Ni-tNTA-E2 + CBU1910-(His)_6_ reaction required optimization to yield unaggregated/precipitated constructs, the details of which are described in the Supporting Information. After conjugation, solutions were purified with size exclusion chromatography (SEC) using a Superose 6 Increase 10/300 GL column (Cytiva) on a FPLC (AKTA, Cytiva). Fractions from the SEC were run on an SDS-PAGE gel and stained with a Pierce Silver Stain Kit (Thermo Fisher Scientific) to determine the presence of CBU1910-E2, E2, and CBU1910, and conjugation efficiencies were estimated by evaluating band intensities with standards. Fractions containing CBU1910-E2 were combined and concentrated with a centrifuge concentrator (Vivaspin 6, 10,000 MWCO). Protein concentration was measured via a bicinchoninic acid assay kit (Pierce). Nanoparticle size was assessed via dynamic light scattering (DLS).

### Construction of SpyTag-E2 Mutants and SpyCatcher-CBU1910 Fusion Protein

Previously established mutants E2(D381C) and E2_152 were used to engineer the SpyTag-E2 platforms. To introduce the D381C mutation to E2_152 via site directed mutagenesis (SDM) the forward primer: 5′-/5Phos/GCCGATCGTTCGTTGCGGTGAAATCGTTGC-3′ and reverse primer: 5′-/5Phos/TTTTCGGCTATACGACCAATACCCAG-3′ were used. Introduction of the SpyTag to E2(D381C) and E2_152 was done using the forward primers: 5′-CATATGGCCCACATCGTTATGGTGGATGCCTACAAGCCAACTAAAGGTTCAGGAACAGCAGGTGGTGGGTCAGGTTCCCTGTCTGTTCCTGGTCCCGC-3′ and 5′-CATATGGCCCACATCGTTATGGTGGATGCCTACAAGCCAACTAAAGCTAGCACCGGCAAAAATGGTCG-3′, respectively. E2 mutants used the same reverse primer: 5′-GGATCCTTAAGCTTCCATCAGCAGCAGTTCCGG-3′.

The plasmid encoding the truncated CBU1910 protein was previously synthesized by GenScript Biotech.^47,69^ The plasmid containing the SpyCatcher gene (pDEST14-SpyCatcher) was obtained from Addgene. To introduce the endonuclease sites and GS-rich spacer on CBU1910 for fusion to SpyCatcher, the forward primer was 5′-GCTAGCGGTTCAGGAACAGCAGGTGGTGGGTCAGGTTCCCCGCAGCAAGTCAAAGACATTC-3′ and the reverse primer was 5′-GGATCCTTATTTTTCGACACGGTCAATTTCTTTTTGCAGG-3′. To introduce the endonuclease sites on SpyCatcher, the forward primer 5′-CATATGTCGTACTACCATCACCATCACCATCACG-3′ and reverse primer 5′-GCTAGCAATATGAGCGTCACCTTTAGTTGCTTTGCC-3′ were used. A standard Phusion High-Fidelity DNA polymerase protocol was used for PCRs. These reactions were performed in a thermal cycler using a 30 s denaturation step at 98 °C, followed by 30 cycles of 15 s at 98 °C, 15 s at 56 °C (SpyTag introduced to E2) or 55 °C (SpyCatcher) or 52 °C (CBU1910), and 45 s (SpyTag introduced to E2) or 40 s (SpyCatcher) or 45 s (CBU1910) at 72 °C, with a final step of 10 min at 72 °C. Sequencing was performed by GeneWiz/Azenta, and DNA and protein sequences are given in Supporting Information.

### Expression, Purification, and Characterization of SpyTag-E2 Particles

The new E2 protein mutants were prepared similarly to previously described mutants.^27,29,30^ Expression analysis of the ST-E2 mutants is described in Supplementary Methods. Mutant ST-E2(D381C) was ultimately chosen for scale up expression. Briefly, a 1 L culture supplemented with 100 μg/mL of ampicillin was inoculated with an overnight culture at 37 °C until an OD of 0.7–0.9 at which time it was induced by 1 mM IPTG and further incubated for 3 h at 37 °C. Cells were pelleted and stored at −80 °C overnight before breaking. Cells were lysed using a lysing buffer containing 3 mM PMSF and French Press (Thermo Fisher Scientific). Soluble cell lysates are heat shocked at 70 °C and ultracentrifuged to remove thermolabile containments. Subsequently, the lysates were purified using a HiPrep Q Sepharose anion exchange column (GE Healthcare) followed by a Superose 6 prep grade (GE Healthcare) size exclusion column. The purified proteins were characterized by DLS (Zetasizer Nano ZS, Malvern), mass spectrometry (Xevo G2-XS QTof) and SDS-PAGE, and bicinchoninic acid assay (BCA) for size, molecular weight and purity, and protein concentration, respectively.

The residual *E. coli* expression derived lipopolysaccharide (LPS) was removed following a previously described method.^27^ Briefly, Triton X-114 (Sigma) was added to the purified protein at 1% (v/v), chilled to 4 °C, vortexed vigorously, and heated to 37 °C. The mixture was then centrifuged at 18000 × *g* and 37 °C for 1 min, and the protein-containing aqueous phase was separated from the detergent phase. This total process was repeated 9 times. Residual Triton was removed with detergent removal spin columns (Pierce). LPS levels were tested to be below 0.1 EU per microgram of E2 protein (LAL ToxinSensor gel clot assay, Genscript).

### Expression, Purification, and Characterization of SpyCatcher-CBU1910

The SpyCatcher-CBU1910 fusion protein was expressed in a fashion similar to that of the E2 particles. Proteins were expressed in *E. coli* via 1 mM IPTG induction. After induction for 3 h at 37 °C, the cells were pelleted and stored at −80 °C before breaking. Cells were lysed via French Press and soluble protein was purified using a HisPur Ni-NTA resin batch protocol (Thermo Fisher Scientific). Briefly, soluble cell lysates were mixed with equal parts equilibration buffer and applied to a HisPur Ni-NTA affinity spin column using a packing ratio of 1.5 mL of resin per 10 mL of lysate slurry. The lysate was allowed to incubate with the resin for 1 h at 4 °C. Wash buffers and elution buffer containing 75 and 150 mM imidazole, and 250 mM imidazole, respectively, were used to attain pure SC-CBU1910. Pure protein fractions were collected and dialyzed into PBS to remove imidazole using 6–8 kDa MWCO dialysis tubing. The purified protein was characterized by mass spectrometry (Xevo G2-XS QTof) and SDS-PAGE, and BCA for molecular weight and purity, and protein concentration, respectively.

Residual *E. coli* expression derived LPS was removed in a similar fashion to the E2 protein. Residual Triton was removed with detergent removal spin columns (Pierce) or SM2 detergent removal beads (Bio-Rad). LPS levels were below 0.1 EU per microgram of SC-CBU1910 protein (LAL ToxinSensor gel clot assay, Genscript).

### CpG and SpyCatcher Conjugation onto SpyTag-E2 Particles

The oligodeoxynucleotide TLR-9 ligand CpG 1826 (5′-tccatgacgttcctgacgtt-3′) (CpG) was synthesized with a phosphorothioated backbone and 5′ benzaldehyde modification by Integrated DNA Technologies (IDT). CpG was conjugated to the internal cavity of the E2 nanoparticle as described previously.^27^ In brief, the internal cavity cysteines of E2 were reduced with TCEP (Pierce) for 30 min, followed by incubation with the *N*-(β-maleimidopropionic acid) hydrazide (BMPH) linker (Pierce) for 2 h at room temperature (RT). Unreacted linker was removed using 40 kDa cutoff Zeba spin desalting columns (Pierce). The aldehyde-modified CpG was subsequently added and incubated overnight at RT. Unreacted CpG was removed by desalting spin columns. Conjugation was estimated by SDS-PAGE and measured by band intensity analysis.^27^

Directly incubating SpyCatcher-CBU1910 and SpyTag-E2 particles allowed for spontaneous isopeptide bond formation and conjugation. SC-CBU1910 proteins were incubated with ST-E2 particles at a ∼0.5:1 (SC-CBU1910:ST-E2 monomer) molar ratio, supplemented with 0.080–0.0875% (w/v) Sarkosyl (SLS), for 20 h at room temperature. SDS-PAGE densitometry analysis with protein standards was used to quantify protein loading onto the particles. DLS and transmission electron microscopy (TEM) were used to measure the size, assembly, and monodispersity of the particles. Transmission electron micrographs of 2% uranyl acetate-stained nanoparticles on Cu 200 or 300 mesh carbon coated grids were obtained on a JEM-2100F (JEOL) instrument with a Gatan OneView camera (Gatan).

Further details describing the optimization trials required to determine the final formulation condition can be found in Supplementary Methods.

### Mice and Immunizations

All animal studies were carried out in accordance with protocols approved by the Institutional Animal Care and Use Committee (IACUC) at the University of California, Irvine. Briefly, 6–8-week-old female C57BL/6 mice (*n* = 5) were immunized subcutaneously at the left flank on Day 0 and followed by a booster on Day 14. Injections were 30 μL per mouse and contained definite amounts of CBU1910, E2, and CpG, based on the formulations investigated. In groups that used the adjuvant, IVAX, an equal volume of IVAX to formulation was supplemented (i.e., 30 μL of E2 formulation + 30 μL IVAX). IVAX contains Addavax (InvivoGen), 1 nmol of CpG 1018, and 3 nmol of MPLA. Seven days after the last immunization, mice were sacrificed, blood was collected via cardiac puncture, and spleens were isolated.

For peptide (CBU1910p) formulations, the same prime boost immunization schedule was followed as described above. Each dosage of the peptide formulation contained 10 μg of CBU1910p and 5 μg of CpG 1826 (when indicated).

### Protein Microarrays

Protein microarrays were fabricated as previously described.^78^ Briefly, CBU1910 protein was diluted to a concentration of 0.1 mg/mL and printed onto nitrocellulose-coated glass Oncyte Avid slides (Grace Bio-Laboratories) using an Omni Grid 100 microarray printer (Genomic Solutions). For probing, mouse plasma samples were diluted 1:100 in protein array blocking buffer supplemented with 10 mg/mL *E. coli* lysate (GenScript) and His-tag containing peptide HHHHHHHHGGGG (Biomatik) to a concentration of 0.1 mg/mL to block anti-polyhistidine antibodies. Arrays were rehydrated with blocking buffer prior to addition of preincubated sera. Arrays were incubated overnight at 4 °C with gentle agitation. After overnight incubation, the slides were washed with Tris-buffered saline (TBS) containing 0.05% Tween 20 (T-TBS) and incubated with biotinylated-SP-conjugated goat anti-mouse IgG, IgG1, or IgG2c (Jackson Immunoresearch). Arrays were washed with T-TBS and incubated with streptavidin conjugated Qdot-800 (ThermoFisher). Arrays were washed three times with T-TBS followed by TBS, dipped in water, and dried by centrifugation. Images were acquired using the ArrayCAM imaging system (Grace Bio-Laboratories). Spot and background intensities were measured using an annotated grid (.gal) file. IgG1 and IgG2c antibody subtype proportions were calculated using respective signal intensities: IgG1/(IgG1 + IgG2c) and IgG2c/(IgG1+IgG2c), respectively.^47^

### T Cell Recall Assays

Recall assays were performed using IFN-γ ELISpot format and spleens collected on day 21 essentially as previously described.^91^ Antigens used for recall were *C. burnetii* CBU1910 and OVA as an irrelevant control antigen. Assays were performed in Roswell Park Memorial Institute (RPMI) 1640, containing 5 × 10^–5^ M β-mercaptoethanol, 100 IU/mL penicillin, 100 μg/mL streptomycin, and 10% heat-inactivated fetal bovine serum (complete medium). Briefly, erythrocyte-depleted splenocytes were incubated at 5 × 10^5^ cells per well in 96-well ELISpot plates, coated previously with IFN-γ capture antibody, and blocked in complete medium, containing titrations of antigen ranging from 2.5 to 10 μg/mL. Mice were assayed separately. Concanavalin A was included as a viability control.

### Statistical Analysis

For nanoparticle characterization, including hydrodynamic diameter measurements, molecular weights determined by mass spectrometry, and antigen/nanoparticle ratios, data are presented as the mean ± standard deviation (S.D.) of at least three independent experiments (*n* ≥ 3), unless otherwise noted. Statistical analysis of immunization data was carried out by using GraphPad Prism. Data are presented as mean ± standard error of the mean (S.E.M.) from at least five independent individuals (*n* ≥ 5). Statistical analysis was determined by a one-way or two-way ANOVA over all groups, followed by a Bonferroni multiple comparison test, unless otherwise noted. *P*-values less than 0.05 were considered significant.

## Supporting Information Available

Supplementary Tables: Abbreviations and description of vaccine components; DNA and protein sequences. Supplementary Figures: Direct recombinant fusion of CBU1910 onto E2 protein nanoparticles; tNTA-Ni + CBU1910 conjugation conditions optimization; Expression and characterization of ST-E2 and SC-CBU1910; Alphafold2 Colab prediction of CBU1910; ST/SC reaction conditions optimization; ELISpot analysis of splenocytes after immunizations; Conjugation of CBU1910p onto E2 nanoparticles; Representative mass spectrometry data for tNTA-E2. Supplementary Methods: Expression analysis of SpyTag-E2 particles; Reaction optimizations to synthesize final ST/SC CBU1910-E2 formulation; Loading *C. burnetii* peptide antigen onto E2 nanoparticles (PDF)
