# Supplementary material for: Engineering Protein Nanoparticles Functionalized with an Immunodominant Coxiella burnetii Antigen to Generate a Q Fever Vaccine
Source: Bioconjug Chem. 2023 Sep 8;34(9):1653–66. doi: 10.1021/acs.bioconjchem.3c00317 (PMC10515490; doi:10.1021/acs.bioconjchem.3c00317)
Supplement: Supplementary file 1 — bc3c00317_si_001.pdf [file bc3c00317_si_001.pdf]

SUPPORTING INFORMATION

Engineering Protein Nanoparticles Functionalized with an Immunodominant *Coxiella burnetii* Antigen to Generate a Q Fever Vaccine

Aaron Ramirez[1], Jiin Felgner[2], Aarti Jain[2], Sharon Jan[2], Tyler J. Albin[3], Alexander J. Badten[1,2], Anthony E. Gregory[2], Rie Nakajima[2], Algimantas Jasinskas[2], Philip L. Felgner[2], Amanda M. Burkhardt[7], D. Huw Davies[2,6,*], Szu-Wen Wang[1,4,5, 6,*]

[1]Department of Chemical and Biomolecular Engineering
[2]Vaccine Research and Development Center, Department of Physiology and Biophysics
[3]Department of Chemistry
[4]Department of Biomedical Engineering
[5]Chao Family Comprehensive Cancer Center
[6]Institute for Immunology
University of California, Irvine, CA 92697, USA

[7]Department of Clinical Pharmacy, School of Pharmacy
University of Southern California
Los Angeles, CA 90089, USA

* Co-corresponding authors:
Szu-Wen Wang (wangsw@uci.edu)
D. Huw Davies (ddavies@uci.edu)

Table SI-1. Abbreviations and description of vaccine components. N/A denotes "not applicable."

**Abbreviation**	**Description**	**Attachment strategy**
CBU1910	*C. burnetii* protein antigen	N/A
E2	E2 protein nanoparticle	N/A
NP	Nanoparticle	N/A
VLP	Virus-like particle	N/A
E2(D381C)	E2 with internal cavity mutation of Asp to Cys	sulfo-SMCC linker
CBU1910p	*C. burnetii* peptide antigen	sulfo-SMCC linker
CBU1910p-E2	CBU1910p attached to E2 nanoparticle via ST/SC	sulfo-SMCC linker
CpG-E2	CpG attached to E2 nanoparticle	sulfo-SMCC linker
CpG-ST-E2	CpG attached to ST-E2 nanoparticle	ST/SC
CBU1910p-CpG-E2	CBU1910p and CpG attached to E2 nanoparticle	sulfo-SMCC linker
E2_152(D381C)	E2 with 20 amino acid N-terminus “tail” and internal cavity mutation of Asp to Cys	Recombinant fusion
E2_158(D381C)	E2 with 14 amino acid N-terminus “tail” and internal cavity mutation of Asp to Cys	Recombinant fusion
CBU1910-E2_152(D381C)	CBU1910 attached to E2_152(D381C) nanoparticle via genetic fusion	Recombinant fusion
CBU1910-E2_158(D381C)	CBU1910 attached to E2_158(D381C) nanoparticle via genetic fusion	Recombinant fusion
E2(E279C)	E2 with external surface mutation of Glu to Cys	Ni-NTA / His
tNTA	Tris-Nitrilotriacetic acid with maleimide functional group	Ni-NTA / His
tNTA-E2	tNTA linker attached to E2(E279C) nanoparticle	Ni-NTA / His
Ni-tNTA-E2	Ni loaded tNTA linker on E2 nanoparticle	Ni-NTA / His
CBU1910-$(His)_6$	Histidine tagged CBU1910	Ni-NTA / His
CBU1910-E2	CBU1910 attached to E2 nanoparticle via Ni-tNTA linker	Ni-NTA / His
ST	SpyTag	ST/SC
SC	SpyCatcher	ST/SC
ST-E2	SpyTag fused to E2 monomer	ST/SC
SC-CBU1910	SpyCatcher fused to CBU1910	ST/SC
CBU1910-E2	CBU1910 attached to E2 nanoparticle via ST/SC	ST/SC
CBU1910-CpG-E2	CBU1910 and CpG attached to E2 nanoparticle	ST/SC

Table SI-2. DNA and protein sequences of E2 nanoparticle mutants and antigen mutants.

Abbreviation	Description: DNA (top) and protein (bottom) sequences	Attachment strategy
**CBU1910**	ccgcagcaagtcaaagacattcagtcaatcgttcatcattacctggtcaatcacc<br>cggaagtcctggttgaagccagccaggcactgcagaagaaaaccgaagctca<br>gcaagaagaacatgctcagcaagcgatcaaagaaaacgcgaaaaaactgttc<br>aacgatccggcatcaccggtcgcaggtaacccgcatggtaatgtcaccctggtgg<br>aatttttcgactatcagtgcggccactgtaaagctatgaattcggtgattcaggcgatc<br>gttaaacagaacaaaaatctgcgtgtggtttttaaagaactgccgattttcggcggtca<br>gagtcaatacgcggccaaagtctccctggcagctgcgaaacagggcaaatattac<br>gcttttcacgatgcgctgctgagcgtggacggtcaactgtctgaacagattaccctgca<br>aacggccgaaaaagttggcctgaacgtcgcacagctgaaaaaagatatggacaat<br>ccggccatccagaaacaactgcgcgataacttccagctggcccaaagtctgcagctg<br>gcaggcaccccgacgtttgtgattggtaacaaagcgctgaccaaattcggtttcattcc<br>gggcgcaacgtcgcaacaaaacctgcaaaaagaaattgaccgtgtcgaaaaataa<br><br>PQQVKDIQSIVHHYLVNHPEVLVEASQALQKKTEAQQEEHAQQ<br>AIKENAKKLFNDPASPVAGNPHGNVTLVEFFDYQCGHCKAMNS<br>VIQAIVKQNKNLRVVFKELPIFGGQSQYAAKVSLAAAKQGKYYA<br>FHDALLSVDGQLSEQITLQTAEKVGLNVAQLKKDMDNPAIQKQL<br>RDNFQLAQSLQLAGTPTFVIGNKALTKFGFIPGATSQQNLQKEI<br>DRVEK	N/A
**E2_152(D381C)**	gctagcaccggcaaaaatggtcgtgtgctgaaagaagacattgatgcgtttct<br>ggcgggcggcgcgaaacccgggcccgctgctgcagaggaaaaggctgctc<br>cagcggctgcgaaaccggctactactgaaggtgaattccctgaaacccgtgaa<br>aaaatgtctggtatccgtcgtgcaatcgcgaaagccatggttcactctaaacaca<br>ccgcgccacacgttaccctgatggatgaagcagacgttaccaaactggttgcgc<br>accgtaaaaaattcaaggcgattgcggcggaaaaaggtatcaaactgaccttcc<br>tgccgtacgttgttaaagctctggtttcggctctgcgtgaatacccggttctgaacac<br>ctctattgacgacgagaccgaagaaatcatccagaaacactactacaacatcgg<br>tatcgctgcggacactgatcgtggtctgctggttcctgtgattaaacacgcggaccg<br>taaaccgatcttcgcgctcgctcaggaaatcaacgaactggctgagaaagctcgtg<br>acggtaaactgactcctggtgaaatgaaaggcgcgtcttgcactattaccaacatcg<br>gctctgcaggtggtcagtggttcaccccagttatcaaccacccggaagttgcgatcc<br>tgggtattggtcgtatagccgaaaagccgatcgttcgt**tgc**ggtgaaatcgttgctgc<br>tccgatgctggccctgtctctgtctttcgatcatcgtatgattgatggcgcgaccgcaca<br>gaaagccctgaaccacatcaaacgtctgctgtccgacccggaactgctgctgatgga<br>agcttaa<br><br>ASTGKNGRVLKEDIDAFLAGGAKPGPAAAEEKAAPAAAKPAT<br>TEGEFPETREKMSGIRRAIAKAMVHSKHTAPHVTLMDEADVT<br>KLVAHRKKFKAIAAEKGIKLTFLPYVVKALVSALREYPVLNTSID<br>DETEEIIQKHYYNIGIAADTDRGLLVPVIKHADRKPIFALAQEIN	Recombinant fusion

	ELAEKARDGKLTPGEMKGASCTITNIGSAGGQWFTPVINHPE<br>VAILGIGRIAEKPIVRCGEIVAAPMLALSLSFDHRMIDGATAQKA<br>LNHIKRLLSDPELLLMEA	
**E2_158(D381C)**	gctagcgtgctgaaagaagacattgatgcgtttctggcgggcggcgcgaaacccgggc<br>ccgctgctgcagaggaaaaggctgctccagcggctgcgaaaccggctactactgaag<br>gtgaattccctgaaacccgtgaaaaaatgtctggtatccgtcgtgcaatcgcgaaagcca<br>tggttcactctaaacacaccgcgccacacgttaccctgatggatgaagcagacgttacca<br>aactggttgcgcaccgtaaaaaattcaaggcgattgcggcggaaaaaggtatcaaact<br>gaccttcctgccgtacgttgttaaagctctggtttcggctctgcgtgaatacccggttctgaac<br>acctctattgacgacgagaccgaagaaatcatccagaaacactactacaacatcggtat<br>cgctgcggacactgatcgtggtctgctggttcctgtgattaaacacgcggaccgtaaacc<br>gatcttcgcgctcgctcaggaaatcaacgaactggctgagaaagctcgtgacggtaaac<br>tgactcctggtgaaatgaaaggcgcgtcttgcactattaccaacatcggctctgcaggtgg<br>tcagtggttcaccccagttatcaaccacccggaagttgcgatcctgggtattggtcgtatag<br>ccgaaaagccgatcgttcgt**tgc**ggtgaaatcgttgctgctccgatgctggccctgtctctg<br>tctttcgatcatcgtatgattgatggcgcgaccgcacagaaagccctgaaccacatcaaa<br>cgtctgctgtccgacccggaactgctgctgatggaagcttaa<br><br>ASVLKEDIDAFLAGGAKPGPAAAEEKAAPAAAKPATTEGEFPET<br>REKMSGIRRAIAKAMVHSKHTAPHVTLMDEADVTKLVAHRKKF<br>KAIAAEKGIKLTFLPYVVKALVSALREYPVLNTSIDDETEEIIQKH<br>YYNIGIAADTDRGLLVPVIKHADRKPIFALAQEINELAEKARDGKL<br>TPGEMKGASCTITNIGSAGGQWFTPVINHPEVAILGIGRIAEKPI<br>VRCGEIVAAPMLALSLSFDHRMIDGATAQKALNHIKRLLSDPELL<br>LMEA	Recombinant fusion
**CBU1910-E2_152(D381C)**	ccgcagcaagtcaaagacattcagtcaatcgttcatcattacctggtcaatcacc<br>cggaagtcctggttgaagccagccaggcactgcagaagaaaaccgaagctca<br>gcaagaagaacatgctcagcaagcgatcaaagaaaacgcgaaaaaactgttc<br>aacgatccggcatcaccggtcgcaggtaacccgcatggtaatgtcaccctggtgg<br>aatttttcgactatcagtgcggccactgtaaagctatgaattcggtgattcaggcgatc<br>gttaaacagaacaaaaatctgcgtgtggtttttaaagaactgccgattttcggcggtca<br>gagtcaatacgcggccaaagtctccctggcagctgcgaaacagggcaaatattac<br>gcttttcacgatgcgctgctgagcgtggacggtcaactgtctgaacagattaccctgca<br>aacggccgaaaaagttggcctgaacgtcgcacagctgaaaaaagatatggacaat<br>ccggccatccagaaacaactgcgcgataacttccagctggcccaaagtctgcagctg<br>gcaggcaccccgacgtttgtgattggtaacaaagcgctgaccaaattcggtttcattcc<br>gggcgcaacgtcgcaacaaaacctgcaaaaagaaattgaccgtgtcgaaaaa<br>gctagcaccggcaaaaatggtcgtgtgctgaaagaagacattgatgcgtttct<br>ggcgggcggcgcgaaacccgggcccgctgctgcagaggaaaaggctgctc<br>cagcggctgcgaaaccggctactactgaaggtgaattccctgaaacccgtgaa<br>aaaatgtctggtatccgtcgtgcaatcgcgaaagccatggttcactctaaacaca<br>ccgcgccacacgttaccctgatggatgaagcagacgttaccaaactggttgcgc	Recombinant fusion

	accgtaaaaaattcaaggcgattgcggcggaaaaaggtatcaaactgaccttcc<br>tgccgtacgttgttaaagctctggtttcggctctgcgtgaatacccggttctgaacac<br>ctctattgacgacgagaccgaagaaatcatccagaaacactactacaacatcgg<br>tatcgctgcggacactgatcgtggtctgctggttcctgtgattaaacacgcggaccg<br>taaaccgatcttcgcgctcgctcaggaaatcaacgaactggctgagaaagctcgtg<br>acggtaaactgactcctggtgaaatgaaaggcgcgtcttgcactattaccaacatcg<br>gctctgcaggtggtcagtggttcaccccagttatcaaccacccggaagttgcgatcc<br>tgggtattggtcgtatagccgaaaagccgatcgttcgt**tgc**ggtgaaatcgttgctgc<br>tccgatgctggccctgtctctgtctttcgatcatcgtatgattgatggcgcgaccgcaca<br>gaaagccctgaaccacatcaaacgtctgctgtccgacccggaactgctgctgatgga<br>agcttaa<br><br>PQQVKDIQSIVHHYLVNHPEVLVEASQALQKKTEAQQEEHAQQ<br>AIKENAKKLFNDPASPVAGNPHGNVTLVEFFDYQCGHCKAMNS<br>VIQAIVKQNKNLRVVFKELPIFGGQSQYAAKVSLAAAKQGKYYA<br>FHDALLSVDGQLSEQITLQTAEKVGLNVAQLKKDMDNPAIQKQL<br>RDNFQLAQSLQLAGTPTFVIGNKALTKFGFIPGATSQQNLQKEI<br>DRVEKASTGKNGRVLKEDIDAFLAGGAKPGPAAAEEKAAPAAA<br>KPATTEGEFPETREKMSGIRRAIAKAMVHSKHTAPHVTLMDEA<br>DVTKLVAHRKKFKAIAAEKGIKLTFLPYVVKALVSALREYPVLNT<br>SIDDETEEIIQKHYYNIGIAADTDRGLLVPVIKHADRKPIFALAQEI<br>NELAEKARDGKLTPGEMKGASCTITNIGSAGGQWFTPVINHPE<br>VAILGIGRIAEKPIVRCGEIVAAPMLALSLSFDHRMIDGATAQKAL<br>NHIKRLLSDPELLLMEA	
**CBU1910-E2_158(D381C)**	ccgcagcaagtcaaagacattcagtcaatcgttcatcattacctggtcaatcacc<br>cggaagtcctggttgaagccagccaggcactgcagaagaaaaccgaagctca<br>gcaagaagaacatgctcagcaagcgatcaaagaaaacgcgaaaaaactgttc<br>aacgatccggcatcaccggtcgcaggtaacccgcatggtaatgtcaccctggtgg<br>aatttttcgactatcagtgcggccactgtaaagctatgaattcggtgattcaggcgatc<br>gttaaacagaacaaaaatctgcgtgtggtttttaaagaactgccgattttcggcggtca<br>gagtcaatacgcggccaaagtctccctggcagctgcgaaacagggcaaatattac<br>gcttttcacgatgcgctgctgagcgtggacggtcaactgtctgaacagattaccctgca<br>aacggccgaaaaagttggcctgaacgtcgcacagctgaaaaaagatatggacaat<br>ccggccatccagaaacaactgcgcgataacttccagctggcccaaagtctgcagctg<br>gcaggcaccccgacgtttgtgattggtaacaaagcgctgaccaaattcggtttcattcc<br>gggcgcaacgtcgcaacaaaacctgcaaaaagaaattgaccgtgtcgaaaaa<br>gctagcgtgctgaaagaagacattgatgcgtttctggcgggcggcgcgaaacccgggc<br>ccgctgctgcagaggaaaaggctgctccagcggctgcgaaaccggctactactgaag<br>gtgaattccctgaaacccgtgaaaaaatgtctggtatccgtcgtgcaatcgcgaaagcca<br>tggttcactctaaacacaccgcgccacacgttaccctgatggatgaagcagacgttacca<br>aactggttgcgcaccgtaaaaaattcaaggcgattgcggcggaaaaaggtatcaaact<br>gaccttcctgccgtacgttgttaaagctctggtttcggctctgcgtgaatacccggttctgaac<br>acctctattgacgacgagaccgaagaaatcatccagaaacactactacaacatcggtat<br>cgctgcggacactgatcgtggtctgctggttcctgtgattaaacacgcggaccgtaaacc<br>gatcttcgcgctcgctcaggaaatcaacgaactggctgagaaagctcgtgacggtaaac	Recombinant fusion

	tgactcctggtgaaatgaaaggcgcgtcttgcactattaccaacatcggctctgcaggtgg<br>tcagtggttcaccccagttatcaaccacccggaagttgcgatcctgggtattggtcgtatag<br>ccgaaaagccgatcgttcgt**tgc**ggtgaaatcgttgctgctccgatgctggccctgtctctg<br>tctttcgatcatcgtatgattgatggcgcgaccgcacagaaagccctgaaccacatcaaa<br>cgtctgctgtccgacccggaactgctgctgatggaagcttaa<br><br>PQQVKDIQSIVHHYLVNHPEVLVEASQALQKKTEAQQEEHAQQ<br>AIKENAKKLFNDPASPVAGNPHGNVTLVEFFDYQCGHCKAMNS<br>VIQAIVKQNKNLRVVFKELPIFGGQSQYAAKVSLAAAKQGKYYA<br>FHDALLSVDGQLSEQITLQTAEKVGLNVAQLKKDMDNPAIQKQL<br>RDNFQLAQSLQLAGTPTFVIGNKALTKFGFIPGATSQQNLQKEI<br>DRVEKASVLKEDIDAFLAGGAKPGPAAAEEKAAPAAAKPATTE<br>GEFPETREKMSGIRRAIAKAMVHSKHTAPHVTLMDEADVTKLV<br>AHRKKFKAIAAEKGIKLTFLPYVVKALVSALREYPVLNTSIDDET<br>EEIIQKHYYNIGIAADTDRGLLVPVIKHADRKPIFALAQEINELAEK<br>ARDGKLTPGEMKGASCTITNIGSAGGQWFTPVINHPEVAILGIG<br>RIAEKPIVRCGEIVAAPMLALSLSFDHRMIDGATAQKALNHIKRL<br>LSDPELLLMEA	
**E2 (E279C)**	atgctgtctgttcctggtcccgctgctgcagaggaaaaggctgctccagcggctgcgaaa<br>ccggctactactgaaggtgaattccctgaaacccgtgaaaaaatgtctggtatccgtcgtg<br>caatcgcgaaagccatggttcactctaaacacaccgcgccacacgttaccctgatggat<br>gaagcagacgttaccaaactggttgcgcaccgtaaaaaattcaaggcgattgcggcgg<br>aaaaaggtatcaaactgaccttcctgccgtacgttgttaaagctctggtttcggctctgcgtg<br>aatacccggttctgaacacctctattgacgactgcaccgaagaaatcatccagaaacact<br>actacaacatcggtatcgctgcggacactgatcgtggtctgctggttcctgtgattaaacac<br>gcggaccgtaaaccgatcttcgcgctcgctcaggaaatcaacgaactggctgagaaag<br>ctcgtgacggtaaactgactcctggtgaaatgaaaggcgcgtcttgcactattaccaacat<br>cggctctgcaggtggtcagtggttcaccccagttatcaaccacccggaagttgcgatcctg<br>ggtattggtcgtatagccgaaaagccgatcgttcgtgacggtgaaatcgttgctgctccgat<br>gctggccctgtctctgtctttcgatcatcgtatgattgatggcgcgaccgcacagaaagccc<br>tgaaccacatcaaacgtctgctgtccgacccggaactgctgctgatggaagcttaa<br><br>MLSVPGPAAAEEKAAPAAAKPATTEGEFPETREKMSGIRRAIAK<br>AMVHSKHTAPHVTLMDEADVTKLVAHRKKFKAIAAEKGIKLTFL<br>PYVVKALVSALREYPVLNTSIDDCTEEIIQKHYYNIGIAADTDRGL<br>LVPVIKHADRKPIFALAQEINELAEKARDGKLTPGEMKGASCTIT<br>NIGSAGGQWFTPVINHPEVAILGIGRIAEKPIVRDGEIVAAPMLAL<br>SLSFDHRMIDGATAQKALNHIKRLLSDPELLLMEA	Ni-NTA / His

**E2 (D381C)**	atgctgtctgttcctggtcccgctgctgcagaggaaaaggctgctccagcggctgc<br>gaaaccggctactactgaaggtgaattccctgaaacccgtgaaaaaatgtctggt<br>atccgtcgtgcaatcgcgaaagccatggttcactctaaacacaccgcgccacac<br>gttaccctgatggatgaagcagacgttaccaaactggttgcgcaccgtaaaaaa<br>ttcaaggcgattgcggcggaaaaaggtatcaaactgaccttcctgccgtacgttgt<br>taaagctctggtttcggctctgcgtgaatacccggttctgaacacctctattgacga<br>cgagaccgaagaaatcatccagaaacactactacaacatcggtatcgctgcgg<br>acactgatcgtggtctgctggttcctgtgattaaacacgcggaccgtaaaccgat<br>cttcgcgctcgctcaggaaatcaacgaactggctgagaaagctcgtgacggta<br>aactgactcctggtgaaatgaaaggcgcgtcttgcactatt<br>accaacatcggctct<br>gcaggtggtcagtggttcaccccagttatcaaccacccggaagttgcgatcctgg<br>gtattggtcgtatagccgaaaagccgatcgttcgt**tgc**ggtgaaatcgttgctgct<br>ccgatgctggccctgtctctgtctttcgatcatcgtatgattgatggcgcgaccgc<br>acagaaagccctgaaccacatcaaacgtctgctgtccgacccggaactgctg<br>ctgatggaagcttaa<br><br>MLSVPGPAAAEEKAAPAAAKPATTEGEFPETREKMSGIRRA<br>IAKAMVHSKHTAPHVTLMDEADVTKLVAHRKKFKAIAAEK<br>GIKLTFLPYVVKALVSALREYPVLNTSIDDETEEIIQKHYYN<br>IGIAADTDRGLLVPVIKHADRKPIFALAQEINELAEKARDGK<br>LTPGEMKGASCTITNIGSAGGQWFTPVINHPEVAILGIGRIA<br>EKPIVRCGEIVAAPMLALSLSFDHRMIDGATAQKALNHIKRL<br>LSDPELLLMEA	ST/SC
**ST**	gcccacatcgttatggtggatgcctacaagccaactaaa<br><br>AHIVMVDAYKPTK	ST/SC
**SC**	atgtcgtactaccatcaccatcaccatcacgattacgacatcccaacgaccg<br>aaaacctgtattttcagggcgccatggttgataccttatcaggtttatcaagtgagca<br>aggtcagtccggtgatatgacaattgaagaagatagtgctacccatattaaattctc<br>aaaacgtgatgaggacggcaaagagttagctggtgcaactatggagttgcgtga<br>ttcatctggtaaaactattagtacatggatttcagatggacaagtgaaagatttctac<br>ctgtatccaggaaaatatacatttgtcgaaaccgcagcaccagacggttatgaggt<br>agcaactgctattacctttacagttaatgagcaaggtcaggttactgtaaatggcaaa<br>gcaactaaaggtgacgctcatatt<br><br>MSYYHHHHHHDYDIPTTENLYFQGAMVDTLSGLSSEQGQS<br>GDMTIEEDSATHIKFSKRDEDGKELAGATMELRDSSGKTIS<br>TWISDGQVKDFYLYPGKYTFVETAAPDGYEVATAITFTVNEQ<br>GQVTVNGKATKGDAHI	ST/SC

**ST-E2**	atggcccacatcgttatggtggatgcctacaagccaactaaaggttcaggaac<br>agcaggtggtgggtcaggttccctgtctgttcctggtcccgctgctgcagaggaaaag<br>gctgctccagcggctgcgaaaccggctactactgaaggtgaattccctgaaacccg<br>tgaaaaaatgtctggtatccgtcgtgcaatcgcgaaagccatggttcactctaaacac<br>accgcgccacacgttaccctgatggatgaagcagacgttaccaaactggttgcgcac<br>cgtaaaaaattcaaggcgattgcggcggaaaaaggtatcaaactgaccttcctgccg<br>tacgttgttaaagctctggtttcggctctgcgtgaatacccggttctgaacacctctattgac<br>gacgagaccgaagaaatcatccagaaacactactacaacatcggtatcgctgcgg<br>acactgatcgtggtctgctggttcctgtgattaaacacgcggaccgtaaaccgatcttc<br>gcgctcgctcaggaaatcaacgaactggctgagaaagctcgtgacggtaaactgac<br>tcctggtgaaatgaaaggcgcgtcttgcactattaccaacatcggctctgcaggtggt<br>cagtggttcaccccagttatcaaccacccggaagttgcgatcctgggtattggtcgtat<br>agccgaaaagccgatcgttcgt**tgc**ggtgaaatcgttgctgctccgatgctggccctg<br>tctctgtctttcgatcatcgtatgattgatggcgcgaccgcacagaaagccctgaacca<br>catcaaacgtctgctgtccgacccggaactgctgctgatggaagcttaa<br><br>MAHIVMVDAYKPTKGSGTAGGGSGSLSVPGPAAAEEKAA<br>PAAAKPATTEGEFPETREKMSGIRRAIAKAMVHSKHTAPHVTL<br>MDEADVTKLVAHRKKFKAIAAEKGIKLTFLPYVVKALVSALRE<br>YPVLNTSIDDETEEIIQKHYYNIGIAADTDRGLLVPVIKHADRK<br>PIFALAQEINELAEKARDGKLTPGEMKGASCTITNIGSAGGQ<br>WFTPVINHPEVAILGIGRIAEKPIVRCGEIVAAPMLALSLSFD<br>HRMIDGATAQKALNHIKRLLSDPELLLMEA	ST/SC
**SC-CBU1910**	atgtcgtactaccatcaccatcaccatcacgattacgacatcccaacgaccg<br>aaaacctgtattttcagggcgccatggttgataccttatcaggtttatcaagtgagca<br>aggtcagtccggtgatatgacaattgaagaagatagtgctacccatattaaattctc<br>aaaacgtgatgaggacggcaaagagttagctggtgcaactatggagttgcgtga<br>ttcatctggtaaaactattagtacatggatttcagatggacaagtgaaagatttctac<br>ctgtatccaggaaaatatacatttgtcgaaaccgcagcaccagacggttatgagg<br>tagcaactgctattacctttacagttaatgagcaaggtcaggttactgtaaatggca<br>aagcaactaaaggtgacgctcatattgctagcggttcaggaacagcaggtggtgg<br>gtcaggttccccgcagcaagtcaaagacattcagtcaatcgttcatcattacctggt<br>caatcacccggaagtcctggttgaagccagccaggcactgcagaagaaaacc<br>gaagctcagcaagaagaacatgctcagcaagcgatcaaagaaaacgcgaaa<br>aaactgttcaacgatccggcatcaccggtcgcaggtaacccgcatggtaatgtca<br>ccctggtggaatttttcgactatcagtgcggccactgtaaagctatgaattcggtgatt<br>caggcgatcgttaaacagaacaaaaatctgcgtgtggtttttaaagaactgccga<br>ttttcggcggtcagagtcaatacgcggccaaagtctccctggcagctgcgaaacag<br>ggcaaatattacgcttttcacgatgcgctgctgagcgtggacggtcaactgtctgaac<br>agattaccctgcaaacggccgaaaaagttggcctgaacgtcgcacagctgaaaaa<br>agatatggacaatccggccatccagaaacaactgcgcgataacttccagctggccc<br>aaagtctgcagctggcaggcaccccgacgtttgtgattggtaacaaagcgctgacca<br>aattcggtttcattccgggcgcaacgtcgcaacaaaacctgcaaaaagaaattgac<br>cgtgtcgaaaaataa	ST/SC

	MSYYHHHHHHDYDIPTTENLYFQGAMVDTLSGLSSEQGQS<br>GDMTIEEDSATHIKFSKRDEDGKELAGATMELRDSSGKTIS<br>TWISDGQVKDFYLYPGKYTFVETAAPDGYEVATAITFTVNE<br>QGQVTVNGKATKGDAHIASGSGTAGGGSGSPQQVKDIQ<br>SIVHHYLVNHPEVLVEASQALQKKTEAQQEEHAQQAIKEN<br>AKKLFNDPASPVAGNPHGNVTLVEFFDYQCGHCKAMNSV<br>IQAIVKQNKNLRVVFKELPIFGGQSQYAAKVSLAAAKQGKY<br>YAFHDALLSVDGQLSEQITLQTAEKVGLNVAQLKKDMDNP<br>AIQKQLRDNFQLAQSLQLAGTPTFVIGNKALTKFGFIPGAT<br>SQQNLQKEIDRVEK	

Supplementary Figures

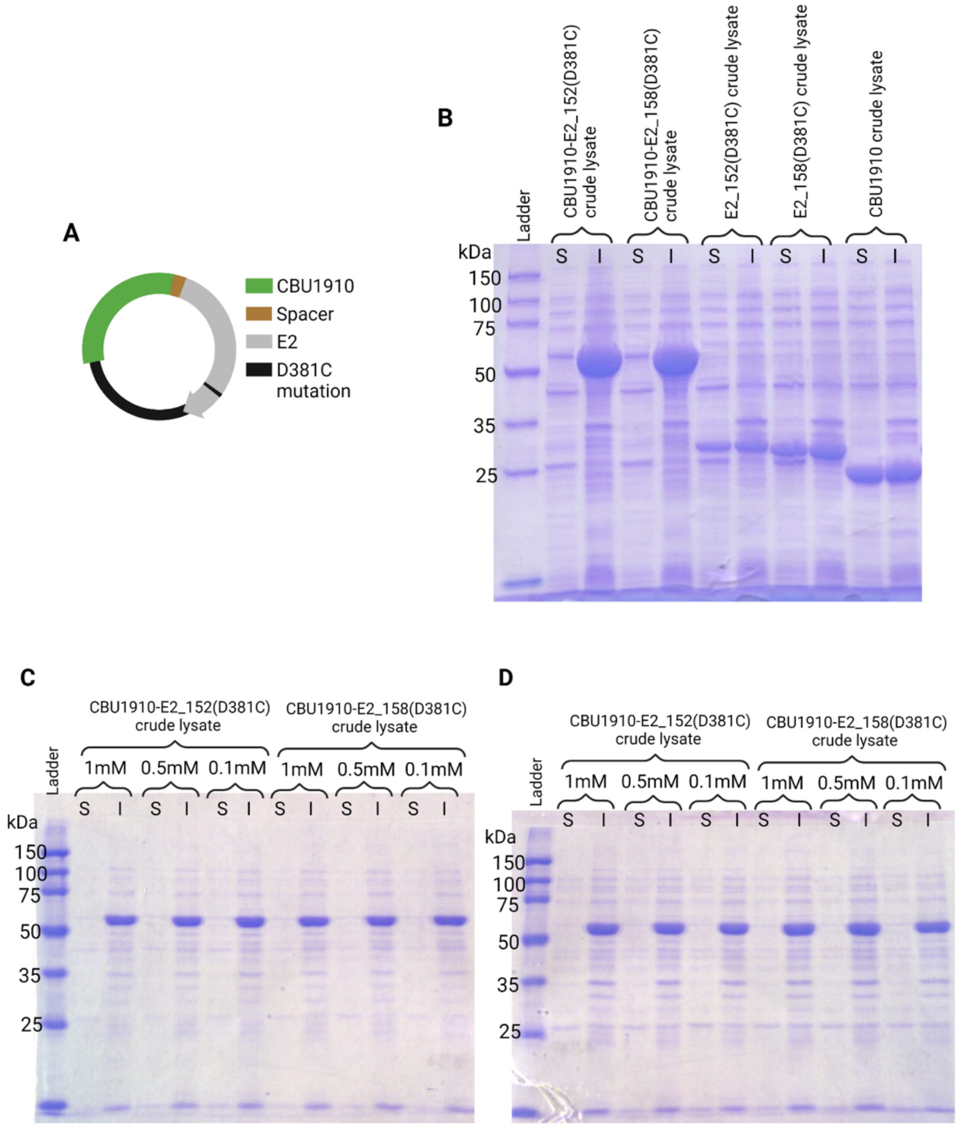

Figure SI-1. Direct recombinant fusion of CBU1910 onto E2 protein nanoparticles. A) Plasmid construction of genetically fused CBU1910-E2 mutant in pET11a vector. B) SDS-PAGE of expression studies of CBU1910-E2 mutants in BL21(DE3) *E. coli*. Expression at 37°C and induced with 1 mM IPTG. S: soluble fraction, I: insoluble fraction C) SDS-PAGE of expression studies at 37°C induced with different concentrations of IPTG. S: soluble fraction, I: insoluble fraction, 1 mM: 1 mM IPTG induction, 0.5 mM: 0.5 mM IPTG induction, 0.1 mM: 1 mM IPTG induction. All samples diluted 1/10 when loaded into SDS-PAGE gel. D) SDS-PAGE of expression studies at 20°C induced with different concentrations of IPTG. S: soluble fraction, I: insoluble fraction, 1 mM: 1 mM IPTG induction, 0.5 mM: 0.5 mM IPTG induction, 0.1 mM: 1 mM IPTG induction.

E2:CBU1910 molar ratio	Buffer	Additive	Concentration of Additive	DLS
1:2	Phosphate buffer (pH = 7.4)	Salt (NaCl)	10 mM	+
			100 mM	+
			200 mM	+
			360 mM	+
1:1	Phosphate buffer (pH = 7.4)	Salt (NaCl)	5 mM	+
			100 mM	+
			200 mM	+
			360 mM	+
1:0.5	Phosphate buffer (pH = 7.4)	Salt (NaCl)	15 mM	+
			100 mM	+
			200 mM	++
			360 mM	++
			450 mM	++
			550 mM	++
		Sucrose	10% (w/v)	+
		Dextrose	5% (w/v)	+
	HEPES buffer (pH = 7.3)	Salt (NaCl)	150 mM	++
			250 mM	+++
			360 mM	++
			450 mM	+++
			550 mM	++
		Sucrose	10% (w/v)	+
		Dextrose	5% (w/v)	+
1:0.3	HEPES buffer (pH = 7.3)	Salt (NaCl)	360 mM	+
		Salt (NaCl) + Tween 80	360mM + 0.004% (v/v)	+
		Salt (NaCl) + Tween 80	360mM + 0.004% (v/v)	+
1:0.1	HEPES buffer (pH = 7.3)	Salt (NaCl)	360 mM	+++
		Salt (NaCl) + Tween 80	360mM + 0.004% (v/v)	+++

Figure SI-2. tNTA-Ni + CBU1910 conjugation conditions optimization. Table of excipients tested to stabilize CBU1910-E2 constructs during tNTA-Ni/HisTag conjugation. Aggregation/precipitation and intact nanoparticle structure were determined based on DLS analysis. +++: high percentage of monodispersed particles; ++: mixture of monodispersed and aggregated particles; +: extreme aggregation of particles. A high NaCl concentration of 360 mM in a HEPES buffer system using low CBU1910:E2 molar ratios demonstrated the most consistent ability to alleviate aggregation during conjugation.

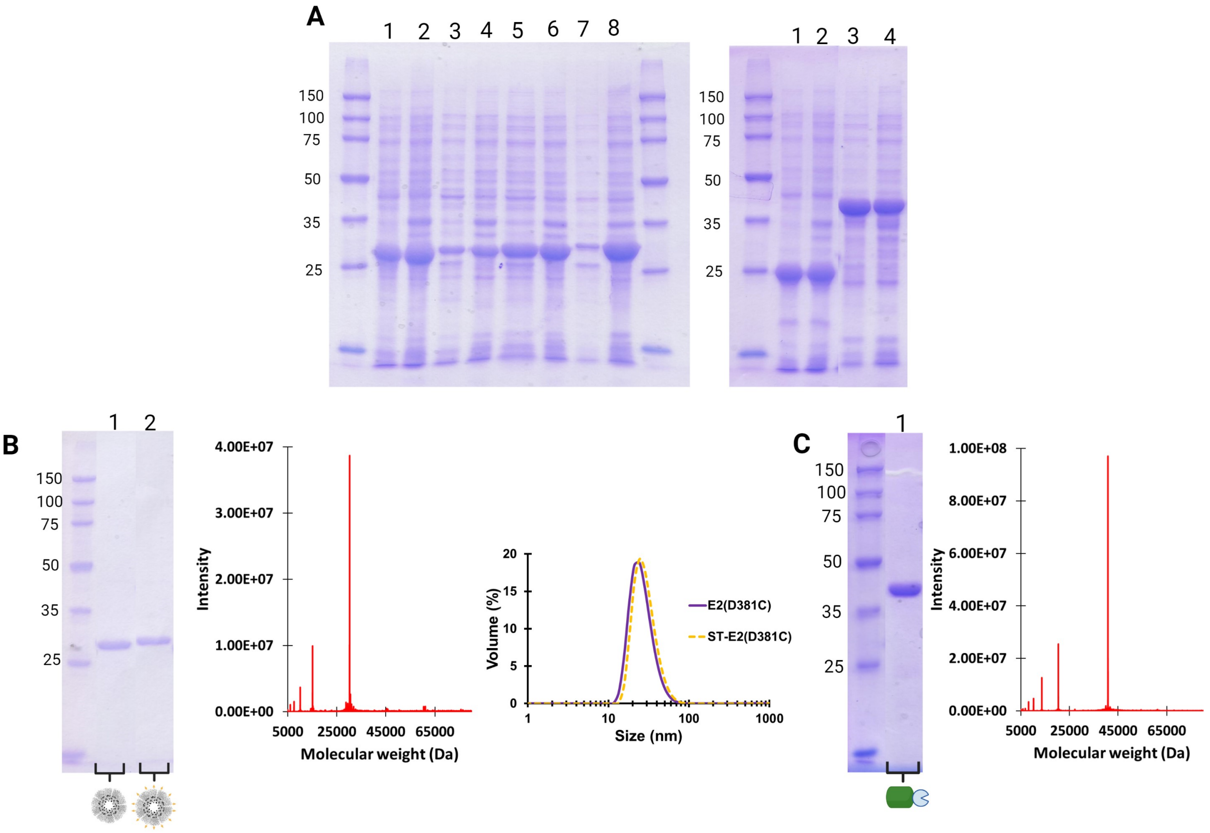

Figure SI-3. Expression and characterization of ST-E2 and SC-CBU1910. A) SDS-PAGE from small scale expression studies of ST-E2 and SC-CBU1910. (left) 1: E2(D381C) soluble fraction 2: E2(D381C) insoluble fraction 3: E2_152(D381C) soluble fraction 4: E2_152(D381C) insoluble fraction 5: ST-E2(D381C) soluble fraction 6: ST-E2(D381C) insoluble fraction 7: ST-E2_152(D381C) soluble fraction 8: ST-E2_152(D381C) insoluble fraction (right) 1: CBU1910 soluble fraction 2: CBU1910 insoluble fraction 3: SC-CBU1910 soluble fraction 4: SC-CBU1910 insoluble fraction. B) Characterization of ST-E2(D381C). Representative (left) SDS-PAGE, (middle) protein mass spectrometry showing ~2 kDa increase in E2 monomer when ST is recombinantly fused to its N-terminus, and (right) DLS indicating ~1 nm increase in diameter when ST is added to surface of E2 nanoparticles. C) Characterization of SC-CBU1910. Representative (left) SDS-PAGE and (right) protein mass spectrometry showing final result of protein-protein with a molecular weight of ~40.8 kDa.

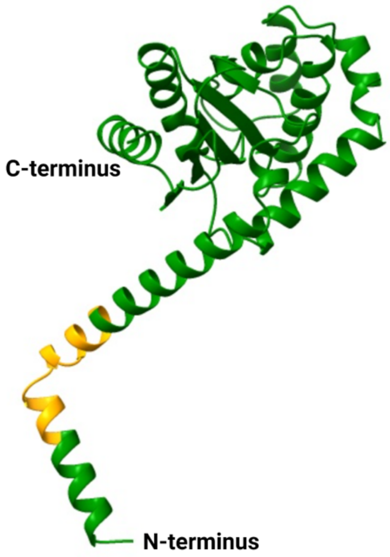

Figure SI-4. Alphafold2 Colab to predict the folded structure of CBU1910. The amino acid sequence of the transmembrane truncated form of CBU1910 was input into Alphafold2 and its output .pdb file was viewed on ChimeraX. Orange highlighted region = CBU1910p sequence.

E2:CBU1910 molar ratio	Buffer	Additive	Concentration of Additive	DLS
1:0.5	Phosphate buffer (pH = 7.4)	None		+
		CHAPS	1% (w/v)	++
			2% (w/v)	++
			10% (w/v)	+
		Tween 20	0.1% (w/v)	+
			1% (w/v)	+
		Tween 80	0.1% (w/v)	+
			1% (w/v)	+
		pH	pH=4-5	+
			pH=9-10	+++X
		Salt (NaCl)	250mM	+
			500mM	++
		SLS	0.1% (w/v)	+++X
			0.09% (w/v)	+++
			0.0875% (w/v)	+++
			0.085% (w/v)	+++
			0.08% (w/v)	+++
			0.0775% (w/v)	++
			0.075% (w/v)	++
			0.0725% (w/v)	+
			0.07% (w/v)	+
			0.0675% (w/v)	++
			0.065% (w/v)	+
			0.06% (w/v)	+
			0.055% (w/v)	+
			0.05% (w/v)	+

Figure SI-5. ST/SC reaction conditions optimization. Table of excipients tested to stabilize CBU1910-E2 constructs during ST/SC reaction. Aggregation/precipitation and intact nanoparticle structure were determined based on SDS-PAGE and DLS analysis. +++X: highly soluble but disassembled particles; +++: high percentage of monodispersed particles; ++: mixture of monodispersed and aggregated particles; +: extreme aggregation of particles. A 0.08-0.0875% (w/v) SLS condition demonstrated consistent ability to alleviate aggregation while keeping nanoparticles intact.

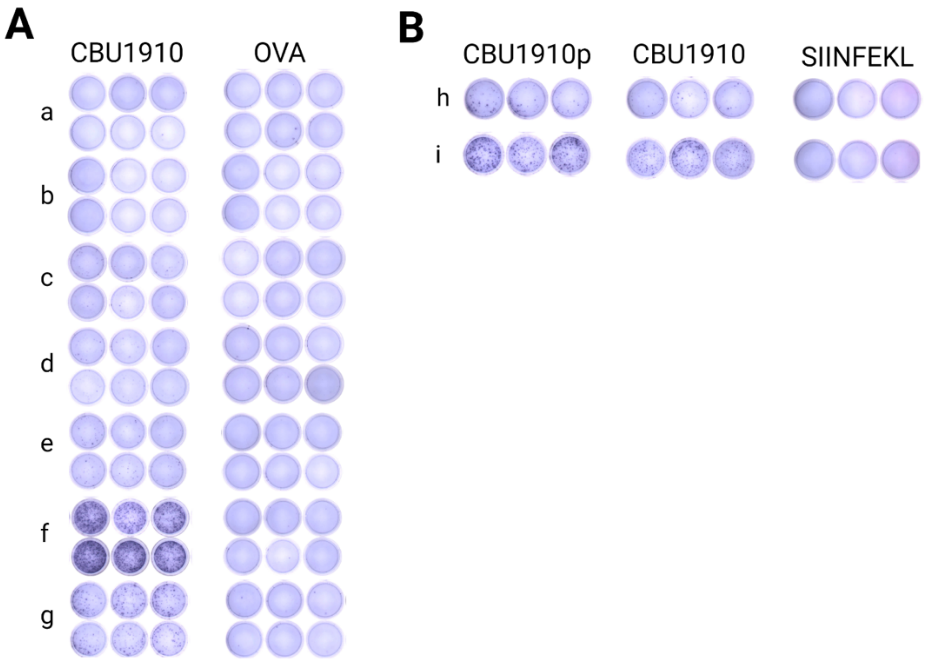

Figure SI-6. ELISpot analysis of splenocytes after immunizations with CBU1910 and CBU1910p formulations. A) Representative ELISpot data from splenocytes of immunized groups (a-g, CBU1910-E2 groups), pulsed ex vivo with relevant protein (CBU1910) or irrelevant protein (OVA) and analyzed for antigen-specific IFN-γ secretion. B) Representative ELISpot data from splenocytes of immunized groups (h = CBU1910p and i = CBU1910p-CpG-E2), pulsed ex vivo with relevant peptide or protein (CBU1910p or CBU1910) or irrelevant peptide (SIINFEKL) and analyzed for antigen-specific IFN-γ secretion.

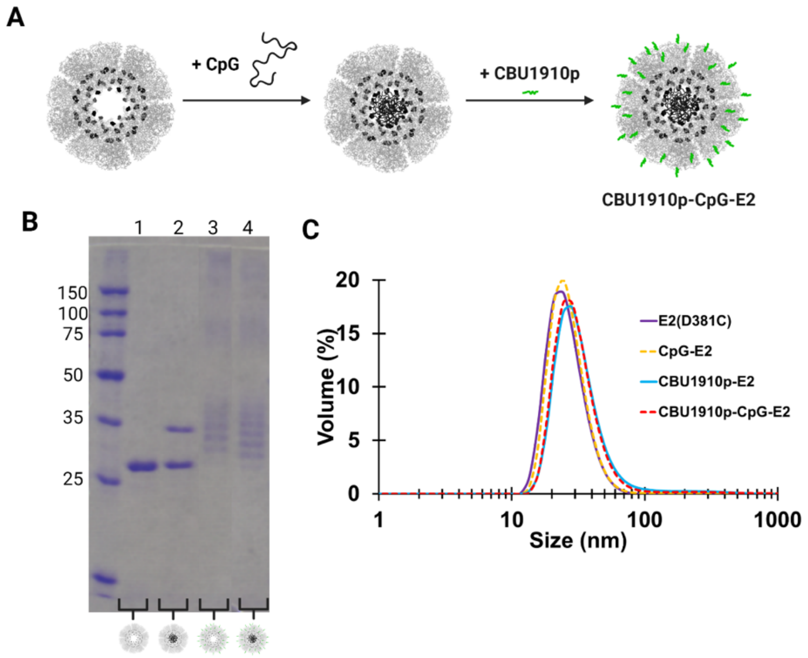

Figure SI-7. Conjugation of CBU1910p onto E2 nanoparticles. A) Structure of E2(D381C) with representative 60 cysteine groups in black. Schematic of CpG1826 and CBU1910p conjugation onto E2. B) SDS-PAGE of formulation synthesis. 1: E2 2: CpG-E2 3: CBU1910p-E2 4: CBU1910p-CpG-E2. C) Hydrodynamic diameters of E2 constructs after CpG and CBU1910p conjugations.

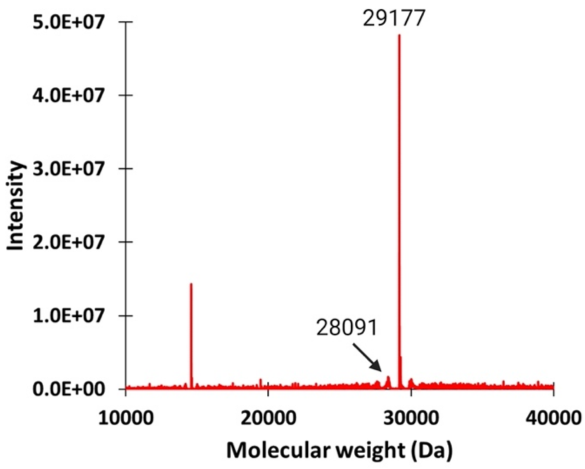

Figure SI-8. Representative mass spectrometry data for E2 monomer conjugated with mal-tNTA (tNTA-E2). E2 monomer (without conjugated tNTA linker) molecular weight is 28091 Da. tNTA-E2 monomer molecular weight is 29177 Da. Data shows near-complete conjugation of the tNTA linker for each protein monomer.

Supplementary Methods

Expression analysis of SpyTag-E2 particles

Expression studies were done for each ST-E2 mutant and controls. Proteins were expressed in BL21 (DE3) *E. coli* via 1 mM IPTG induction. After induction for 3 h at 37 °C, cells were pelleted and stored at -80 °C before breaking. Cells were lysed by vortexing with glass beads. Soluble and insoluble lysates were centrifuged at 18000 x g for 15 min and analyzed using SDS-PAGE for molecular weight and soluble: insoluble ratios. Mutant ST-E2(D381C) was chosen for subsequent scale up expression.

Reaction optimizations to synthesize final ST/SC CBU1910-E2 formulation.

Investigated excipients included Sarkosyl (SLS) concentrations from 0.05-5.0% (w/v), 3-((3-cholamidopropyl) dimethylammonio)-1-propanesulfonate (CHAPS) concentrations from 1.0-10% (w/v), Tween 20 concentrations from 0.10-1% (w/v), Tween 80 concentrations from 0.10-1% (w/v), pH ranges from 5-10, NaCl concentrations from 100-500 mM, and dextrose concentrations from 5.0-10% (w/v). Molar ratios of SC-CBU1910:ST-E2 monomer ranged from 0.5-3. Reaction temperatures and times ranged from 4-25 °C and 30 min-24 h, respectively. The soluble and insoluble fractions of these reactions were separated by centrifuging at 18000 x g for 5 min and analyzed using DLS and SDS-PAGE densitometry.

Loading *C. burnetii* peptide antigen onto E2 nanoparticles

Given the desire to elicit a robust adaptive immune response towards the pathogen of *C. burnetii*, we conjugated peptide antigen epitopes to the E2 nanoparticle, a strategy which has been implemented by our cancer vaccines.[1-3] Using a CBU1910 peptide, HYLVNHPEVLVEASQ (CBU1910p), that has been shown to be a T cell specific epitope, the CBU1910p-CpG-E2 formulation was synthesized.[4,5] Optimization of the peptide conjugation was necessary to synthesize well loaded and stable constructs. To increase its physical stability and prevent aggregation, the final formulation contained 0.005% (v/v) Tween 20. Conjugation of CBU1910p was supported by ~1.8 kDa incremental

increases to E2 monomer molecular weight seen on SDS-PAGE (Figure SI-7). Using a BMPH linker, aldehyde modified CpG was conjugated in the core of the E2 nanoparticle. Loading of CpG was confirmed by a ~7 kDa increase in E2 monomer molecular weight from ~28 kDa to ~35 kDa. SDS-PAGE and mass spectrometry were used to determine the loading of CBU1910p and CpG per nanoparticle. Quantification indicated 22.3 ± 1.5 CpG 1826 molecules were conjugated internally and 166 ± 11.2 CBU1010p peptides were conjugated externally per 60-mer E2 nanoparticle, similar to previous E2 formulations.[1] The average hydrodynamic diameter of CpG-E2, CBU1910p-E2 and CBU1910p-CpG-E2 nanoparticles was 27.1 ± 0.4 nm, 35.4 ± 2.7 nm, and 31.9 ± 1.7 nm, respectively (Figure SI-7).

References

1. Molino, N. M.; Anderson, A. K. L.; Nelson, E. L.; Wang, S.-W., Biomimetic Protein Nanoparticles Facilitate Enhanced Dendritic Cell Activation and Cross-Presentation. *ACS Nano* **2013,** *7* (11), 9743-9752.
2. Molino, N. M.; Neek, M.; Tucker, J. A.; Nelson, E. L.; Wang, S.-W., Viral-mimicking protein nanoparticle vaccine for eliciting anti-tumor responses. *Biomaterials* **2016,** *86*, 83-91.
3. Neek, M.; Tucker, J. A.; Kim, T. I.; Molino, N. M.; Nelson, E. L.; Wang, S.-W., Co-delivery of human cancer-testis antigens with adjuvant in protein nanoparticles induces higher cell-mediated immune responses. *Biomaterials* **2018,** *156*, 194-203.
4. Chen, C.; Dow, C.; Wang, P.; Sidney, J.; Read, A.; Harmsen, A.; Samuel, J. E.; Peters, B., Identification of CD4+ T Cell Epitopes in C. burnetii Antigens Targeted by Antibody Responses. **2011,** *6* (3), e17712.
5. Xiong, X.; Qi, Y.; Jiao, J.; Gong, W.; Duan, C.; Wen, B., Exploratory Study on Th1 Epitope-Induced Protective Immunity against Coxiella burnetii Infection. *PLoS ONE* **2014,** *9* (1), e87206.
